# Supplementary material for: Spatiotemporal dynamics of ethylene biosynthesis shape infection and nodule initiation in Medicago truncatula
Source: Plant Cell. 2026 Jun 10;38(6):koag173. doi: 10.1093/plcell/koag173 (PMC13291814; doi:10.1093/plcell/koag173)
Supplement: koag173_Supplementary_Data [file koag173_supplementary_data.zip › Supplemental file S1.pdf]

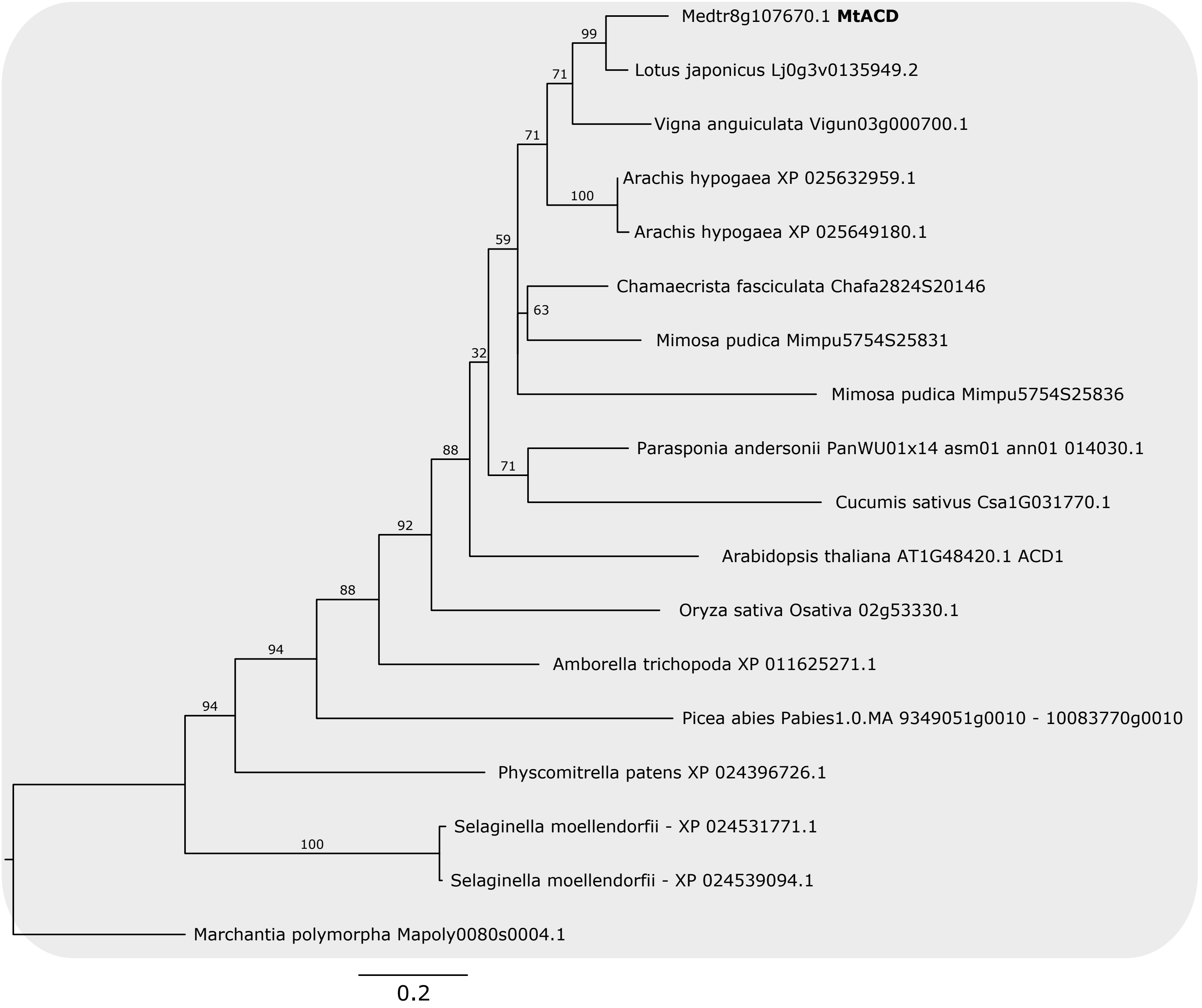

Medtr8g107670.1 **MtACD**
Lotus japonicus Lj0g3v0135949.2
Vigna anguiculata Vigun03g000700.1
Arachis hypogaea XP 025632959.1
Arachis hypogaea XP 025649180.1
Chamaecrista fasciculata Chafa2824S20146
Mimosa pudica Mimpu5754S25831
Mimosa pudica Mimpu5754S25836
Parasponia andersonii PanWU01x14 asm01 ann01 014030.1
Cucumis sativus Csa1G031770.1
Arabidopsis thaliana AT1G48420.1 ACD1
Oryza sativa Osativa 02g53330.1
Amborella trichopoda XP 011625271.1
Picea abies Pabies1.0.MA 9349051g0010 - 10083770g0010
Physcomitrella patens XP 024396726.1
Selaginella moellendorfii - XP 024531771.1
Selaginella moellendorfii - XP 024539094.1
Marchantia polymorpha Mapoly0080s0004.1
99
71
71
100
59
63
32
88
71
92
88
94
94
100
0.2

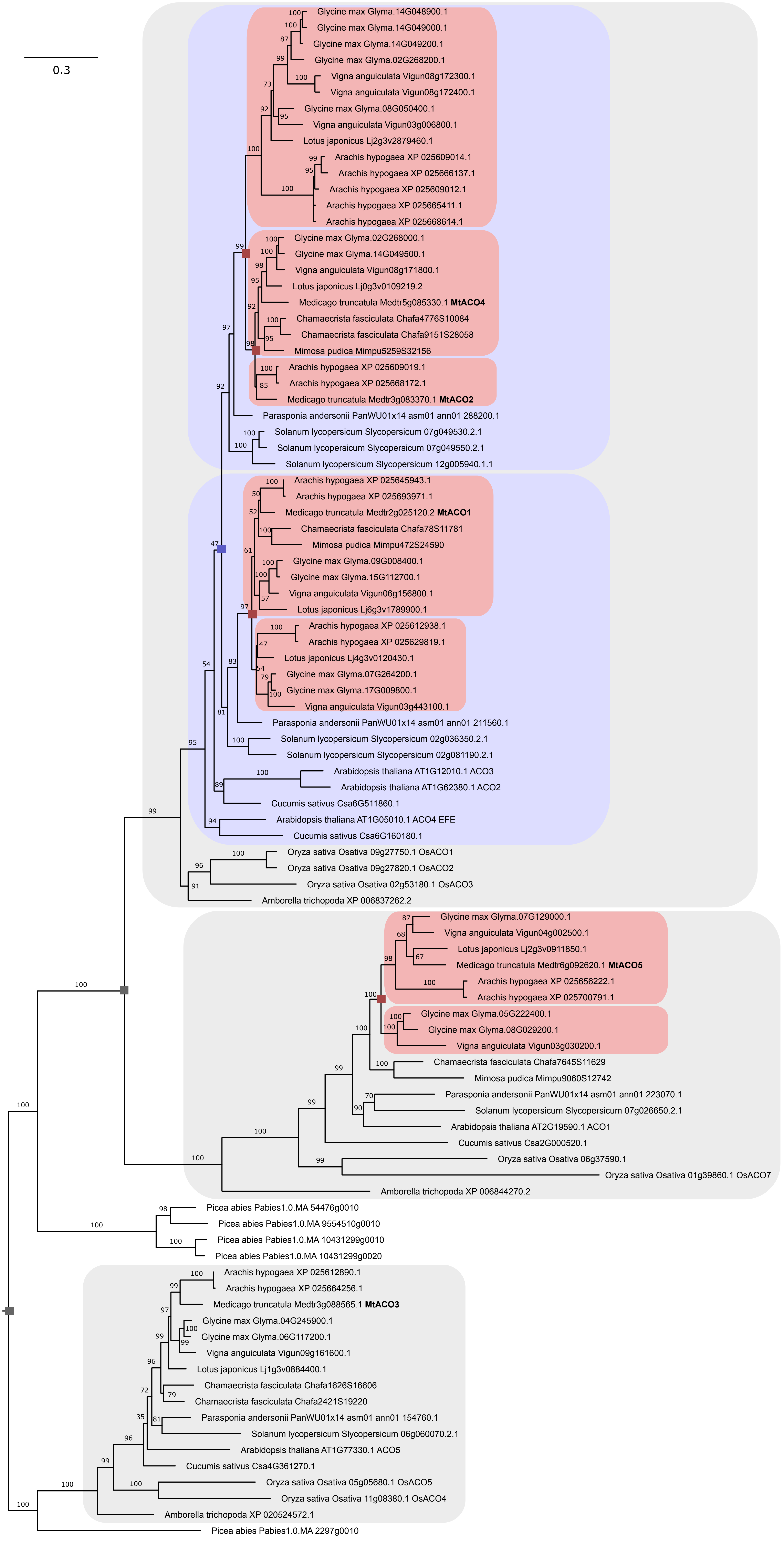

0.3
Glycine max Glyma.14G048900.1
Glycine max Glyma.14G049000.1
Glycine max Glyma.14G049200.1
Glycine max Glyma.02G268200.1
Vigna anguiculata Vigun08g172300.1
Vigna anguiculata Vigun08g172400.1
Glycine max Glyma.08G050400.1
Vigna anguiculata Vigun03g006800.1
Lotus japonicus Lj2g3v2879460.1
Arachis hypogaea XP 025609014.1
Arachis hypogaea XP 025666137.1
Arachis hypogaea XP 025609012.1
Arachis hypogaea XP 025665411.1
Arachis hypogaea XP 025668614.1
Glycine max Glyma.02G268000.1
Glycine max Glyma.14G049500.1
Vigna anguiculata Vigun08g171800.1
Lotus japonicus Lj0g3v0109219.2
Medicago truncatula Medtr5g085330.1 **MtACO4**
Chamaecrista fasciculata Chafa4776S10084
Chamaecrista fasciculata Chafa9151S28058
Mimosa pudica Mimpu5259S32156
Arachis hypogaea XP 025609019.1
Arachis hypogaea XP 025668172.1
Medicago truncatula Medtr3g083370.1 **MtACO2**
Parasponia andersonii PanWU01x14 asm01 ann01 288200.1
Solanum lycopersicum Slycopersicum 07g049530.2.1
Solanum lycopersicum Slycopersicum 07g049550.2.1
Solanum lycopersicum Slycopersicum 12g005940.1.1
Arachis hypogaea XP 025645943.1
Arachis hypogaea XP 025693971.1
Medicago truncatula Medtr2g025120.2 **MtACO1**
Chamaecrista fasciculata Chafa78S11781
Mimosa pudica Mimpu472S24590
Glycine max Glyma.09G008400.1
Glycine max Glyma.15G112700.1
Vigna anguiculata Vigun06g156800.1
Lotus japonicus Lj6g3v1789900.1
Arachis hypogaea XP 025612938.1
Arachis hypogaea XP 025629819.1
Lotus japonicus Lj4g3v0120430.1
Glycine max Glyma.07G264200.1
Glycine max Glyma.17G009800.1
Vigna anguiculata Vigun03g443100.1
Parasponia andersonii PanWU01x14 asm01 ann01 211560.1
Solanum lycopersicum Slycopersicum 02g036350.2.1
Solanum lycopersicum Slycopersicum 02g081190.2.1
Arabidopsis thaliana AT1G12010.1 ACO3
Arabidopsis thaliana AT1G62380.1 ACO2
Cucumis sativus Csa6G511860.1
Arabidopsis thaliana AT1G05010.1 ACO4 EFE
Cucumis sativus Csa6G160180.1
Oryza sativa Osativa 09g27750.1 OsACO1
Oryza sativa Osativa 09g27820.1 OsACO2
Oryza sativa Osativa 02g53180.1 OsACO3
Amborella trichopoda XP 006837262.2
Glycine max Glyma.07G129000.1
Vigna anguiculata Vigun04g002500.1
Lotus japonicus Lj2g3v0911850.1
Medicago truncatula Medtr6g092620.1 **MtACO5**
Arachis hypogaea XP 025656222.1
Arachis hypogaea XP 025700791.1
Glycine max Glyma.05G222400.1
Glycine max Glyma.08G029200.1
Vigna anguiculata Vigun03g030200.1
Chamaecrista fasciculata Chafa7645S11629
Mimosa pudica Mimpu9060S12742
Parasponia andersonii PanWU01x14 asm01 ann01 223070.1
Solanum lycopersicum Slycopersicum 07g026650.2.1
Arabidopsis thaliana AT2G19590.1 ACO1
Cucumis sativus Csa2G000520.1
Oryza sativa Osativa 06g37590.1
Oryza sativa Osativa 01g39860.1 OsACO7
Amborella trichopoda XP 006844270.2
Picea abies Pabies1.0.MA 54476g0010
Picea abies Pabies1.0.MA 9554510g0010
Picea abies Pabies1.0.MA 10431299g0010
Picea abies Pabies1.0.MA 10431299g0020
Arachis hypogaea XP 025612890.1
Arachis hypogaea XP 025664256.1
Medicago truncatula Medtr3g088565.1 **MtACO3**
Glycine max Glyma.04G245900.1
Glycine max Glyma.06G117200.1
Vigna anguiculata Vigun09g161600.1
Lotus japonicus Lj1g3v0884400.1
Chamaecrista fasciculata Chafa1626S16606
Chamaecrista fasciculata Chafa2421S19220
Parasponia andersonii PanWU01x14 asm01 ann01 154760.1
Solanum lycopersicum Slycopersicum 06g060070.2.1
Arabidopsis thaliana AT1G77330.1 ACO5
Cucumis sativus Csa4G361270.1
Oryza sativa Osativa 05g05680.1 OsACO5
Oryza sativa Osativa 11g08380.1 OsACO4
Amborella trichopoda XP 020524572.1
Picea abies Pabies1.0.MA 2297g0010

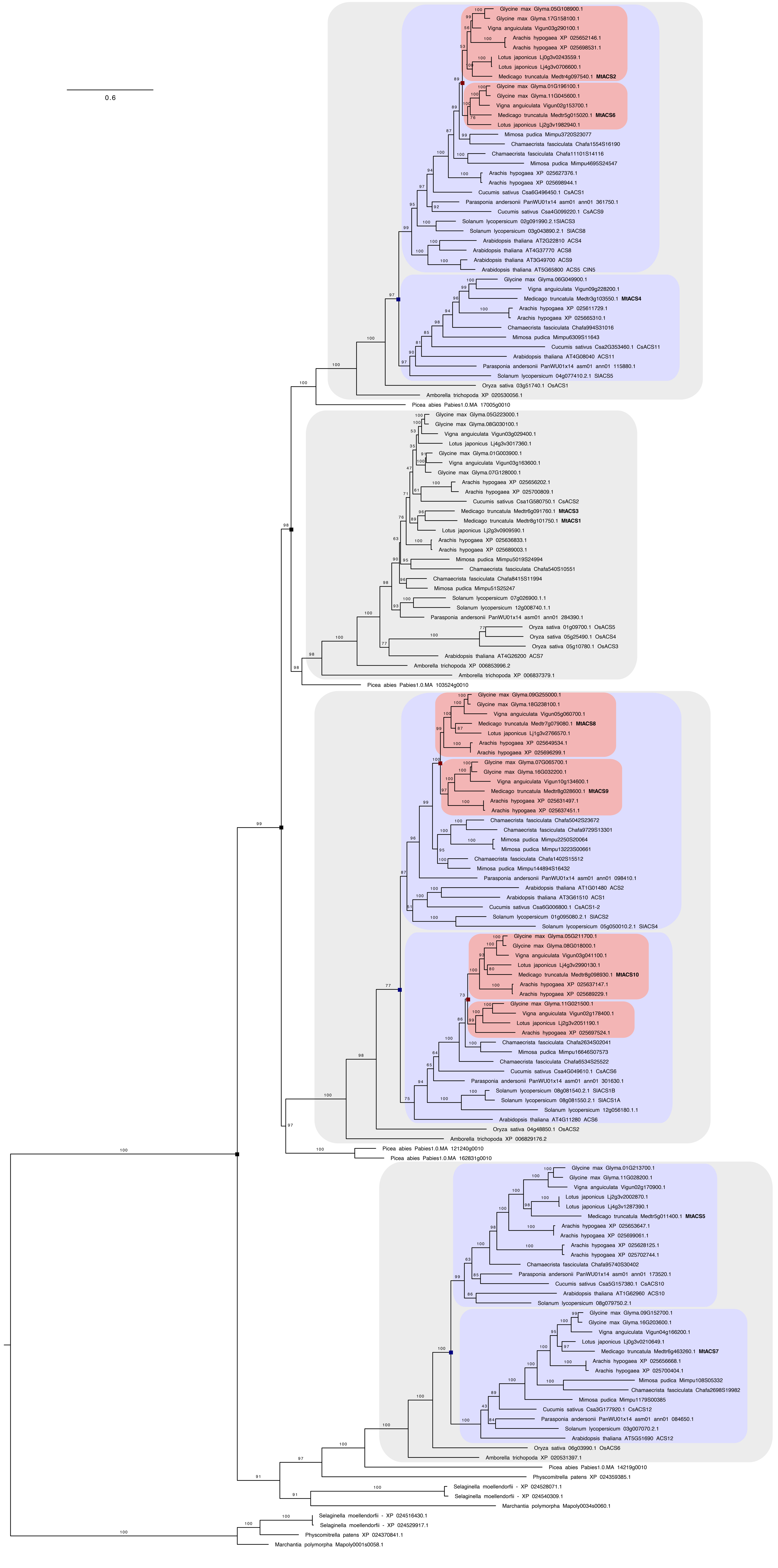
0.6
Glycine max Glyma.05G108900.1
Glycine max Glyma.17G158100.1
Vigna anguiculata Vigun03g290100.1
Arachis hypogaea XP 025652146.1
Arachis hypogaea XP 025698531.1
Lotus japonicus Lj0g3v0243559.1
Lotus japonicus Lj4g3v0706600.1
Medicago truncatula Medtr4g097540.1 MtACS2
Glycine max Glyma.01G196100.1
Glycine max Glyma.11G045600.1
Vigna anguiculata Vigun02g153700.1
Medicago truncatula Medtr5g015020.1 MtACS6
Lotus japonicus Lj2g3v1982940.1
Mimosa pudica Mimpu3720S23077
Chamaecrista fasciculata Chafa1554S16190
Chamaecrista fasciculata Chafa11101S14116
Mimosa pudica Mimpu4695S24547
Arachis hypogaea XP 025627376.1
Arachis hypogaea XP 025698944.1
Cucumis sativus Csa6G496450.1 CsACS1
Parasponia andersonii PanWU01x14 asm01 ann01 361750.1
Cucumis sativus Csa4G099220.1 CsACS9
Solanum lycopersicum 02g091990.2.1SlACS3
Solanum lycopersicum 03g043890.2.1 SlACS8
Arabidopsis thaliana AT2G22810 ACS4
Arabidopsis thaliana AT4G37770 ACS8
Arabidopsis thaliana AT3G49700 ACS9
Arabidopsis thaliana AT5G65800 ACS5 CIN5
Glycine max Glyma.06G049900.1
Vigna anguiculata Vigun09g228200.1
Medicago truncatula Medtr3g103550.1 MtACS4
Arachis hypogaea XP 025611729.1
Arachis hypogaea XP 025665310.1
Chamaecrista fasciculata Chafa994S31016
Mimosa pudica Mimpu6309S11643
Cucumis sativus Csa2G353460.1 CsACS11
Arabidopsis thaliana AT4G08040 ACS11
Parasponia andersonii PanWU01x14 asm01 ann01 115880.1
Solanum lycopersicum 04g077410.2.1 SlACS5
Oryza sativa 03g51740.1 OsACS1
Amborella trichopoda XP 020530056.1
Picea abies Pabies1.0.MA 17005g0010
Glycine max Glyma.05G223000.1
Glycine max Glyma.08G030100.1
Vigna anguiculata Vigun03g029400.1
Lotus japonicus Lj4g3v3017360.1
Glycine max Glyma.01G003900.1
Vigna anguiculata Vigun03g163600.1
Glycine max Glyma.07G128000.1
Arachis hypogaea XP 025656202.1
Arachis hypogaea XP 025700809.1
Cucumis sativus Csa1G580750.1 CsACS2
Medicago truncatula Medtr6g091760.1 MtACS3
Medicago truncatula Medtr8g101750.1 MtACS1
Lotus japonicus Lj2g3v0909590.1
Arachis hypogaea XP 025636833.1
Arachis hypogaea XP 025689003.1
Mimosa pudica Mimpu5019S24994
Chamaecrista fasciculata Chafa540S10551
Chamaecrista fasciculata Chafa8415S11994
Mimosa pudica Mimpu51S25247
Solanum lycopersicum 07g026900.1.1
Solanum lycopersicum 12g008740.1.1
Parasponia andersonii PanWU01x14 asm01 ann01 284390.1
Oryza sativa 01g09700.1 OsACS5
Oryza sativa 05g25490.1 OsACS4
Oryza sativa 05g10780.1 OsACS3
Arabidopsis thaliana AT4G26200 ACS7
Amborella trichopoda XP 006853996.2
Amborella trichopoda XP 006837379.1
Picea abies Pabies1.0.MA 103524g0010
Glycine max Glyma.09G255000.1
Glycine max Glyma.18G238100.1
Vigna anguiculata Vigun05g060700.1
Medicago truncatula Medtr7g079080.1 MtACS8
Lotus japonicus Lj1g3v2766570.1
Arachis hypogaea XP 025649534.1
Arachis hypogaea XP 025696299.1
Glycine max Glyma.07G065700.1
Glycine max Glyma.16G032200.1
Vigna anguiculata Vigun10g134600.1
Medicago truncatula Medtr8g028600.1 MtACS9
Arachis hypogaea XP 025631497.1
Arachis hypogaea XP 025637451.1
Chamaecrista fasciculata Chafa5042S23672
Chamaecrista fasciculata Chafa9729S13301
Mimosa pudica Mimpu2250S20064
Mimosa pudica Mimpu13223S00661
Chamaecrista fasciculata Chafa1402S15512
Mimosa pudica Mimpu144894S16432
Parasponia andersonii PanWU01x14 asm01 ann01 098410.1
Arabidopsis thaliana AT1G01480 ACS2
Arabidopsis thaliana AT3G61510 ACS1
Cucumis sativus Csa6G006800.1 CsACS1-2
Solanum lycopersicum 01g095080.2.1 SlACS2
Solanum lycopersicum 05g050010.2.1 SlACS4
Glycine max Glyma.05G211700.1
Glycine max Glyma.08G018000.1
Vigna anguiculata Vigun03g041100.1
Lotus japonicus Lj4g3v2990130.1
Medicago truncatula Medtr8g098930.1 MtACS10
Arachis hypogaea XP 025637147.1
Arachis hypogaea XP 025689229.1
Glycine max Glyma.11G021500.1
Vigna anguiculata Vigun02g178400.1
Lotus japonicus Lj2g3v2051190.1
Arachis hypogaea XP 025697524.1
Chamaecrista fasciculata Chafa2634S02041
Mimosa pudica Mimpu16646S07573
Chamaecrista fasciculata Chafa6534S25522
Cucumis sativus Csa4G049610.1 CsACS6
Parasponia andersonii PanWU01x14 asm01 ann01 301630.1
Solanum lycopersicum 08g081540.2.1 SlACS1B
Solanum lycopersicum 08g081550.2.1 SlACS1A
Solanum lycopersicum 12g056180.1.1
Arabidopsis thaliana AT4G11280 ACS6
Oryza sativa 04g48850.1 OsACS2
Amborella trichopoda XP 006829176.2
Picea abies Pabies1.0.MA 121240g0010
Picea abies Pabies1.0.MA 162831g0010
Glycine max Glyma.01G213700.1
Glycine max Glyma.11G028200.1
Vigna anguiculata Vigun02g170900.1
Lotus japonicus Lj2g3v2002870.1
Lotus japonicus Lj4g3v1287390.1
Medicago truncatula Medtr5g011400.1 MtACS5
Arachis hypogaea XP 025653647.1
Arachis hypogaea XP 025699061.1
Arachis hypogaea XP 025628125.1
Arachis hypogaea XP 025702744.1
Chamaecrista fasciculata Chafa95740S30402
Parasponia andersonii PanWU01x14 asm01 ann01 173520.1
Cucumis sativus Csa5G157380.1 CsACS10
Arabidopsis thaliana AT1G62960 ACS10
Solanum lycopersicum 08g079750.2.1
Glycine max Glyma.09G152700.1
Glycine max Glyma.16G203600.1
Vigna anguiculata Vigun04g166200.1
Lotus japonicus Lj0g3v0210649.1
Medicago truncatula Medtr6g463260.1 MtACS7
Arachis hypogaea XP 025656668.1
Arachis hypogaea XP 025700404.1
Mimosa pudica Mimpu108S05332
Chamaecrista fasciculata Chafa2698S19982
Mimosa pudica Mimpu1179S00385
Cucumis sativus Csa3G177920.1 CsACS12
Parasponia andersonii PanWU01x14 asm01 ann01 084650.1
Solanum lycopersicum 03g007070.2.1
Arabidopsis thaliana AT5G51690 ACS12
Oryza sativa 06g03990.1 OsACS6
Amborella trichopoda XP 020531397.1
Picea abies Pabies1.0.MA 14219g0010
Physcomitrella patens XP 024359385.1
Selaginella moellendorffii - XP 024528071.1
Selaginella moellendorffii - XP 024540309.1
Marchantia polymorpha Mapoly0034s0060.1
Selaginella moellendorffii - XP 024516430.1
Selaginella moellendorffii - XP 024529917.1
Physcomitrella patens XP 024370841.1
Marchantia polymorpha Mapoly0001s0058.1

Supplemental file S1. Full phylogenetic trees of *ACD*, *ACO,* and *ACS* protein sequences. Paralogous orthogroups specific to flowering plants are indicated in grey; those specific to Eudicots in blue; those specific to legumes in red.
