# Supplementary material for: Spatiotemporal dynamics of ethylene biosynthesis shape infection and nodule initiation in Medicago truncatula
Source: Plant Cell. 2026 Jun 10;38(6):koag173. doi: 10.1093/plcell/koag173 (PMC13291814; doi:10.1093/plcell/koag173)
Supplement: koag173_Supplementary_Data [file koag173_supplementary_data.zip › Supplementary Data.pdf]

Supplemental Figures to:

Spatiotemporal dynamics of ethylene biosynthesis shape infection and nodule initiation in *Medicago truncatula*

Sophia Müller[1,#], Thijs Stegmann[1,#], Kelvin Adema[1], Rens Holmer[2,^], Amber van Seters[1], Robin van Velzen[3], Olga Kulikova[2], Tristan Wijsman[1], Joel Klein[2,$], Josefina-Patricia Fernandez-Moreno[4], Anna N. Stepanova[4], Jose M. Alonso[4], Henk Franssen[2], Estibaliz Larrainzar[5], Arjan van Zeijl[2,‡], Wouter Kohlen[1,2,*]

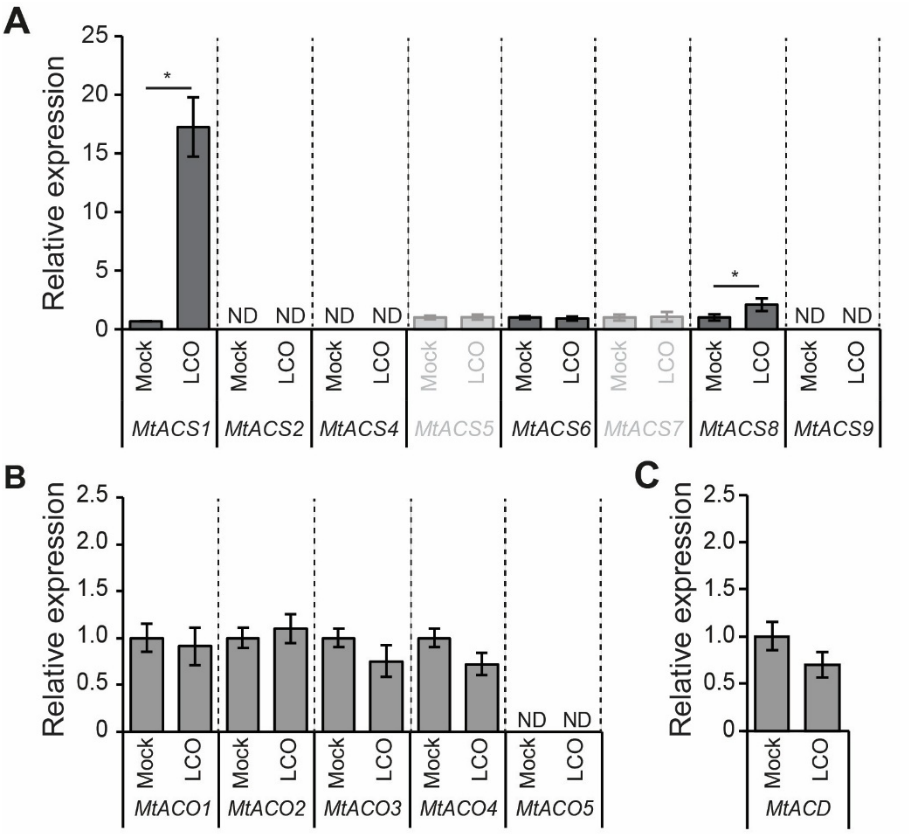

Supplemental Figure S1. Relative expression of ethylene biosynthesis and ACC metabolism gene *following mock and lipo-chitooligosaccharide (LCO) application, measured at 3 hours post-treatment of (A) MtACS1, 2, 4, 5, 6, 7, 8, 9, (B) MtACO1, 2, 3, 4, 5, and (C) MtACD*. Bars represent mean ± SE (n=3, independent biological replicates each consisting of the susceptible zones of ~16 pooled plate grown plants). Asterisks (*) indicate significant differences between treatments (Student t-test, $P < 0.05$) (Supports Figure 2).

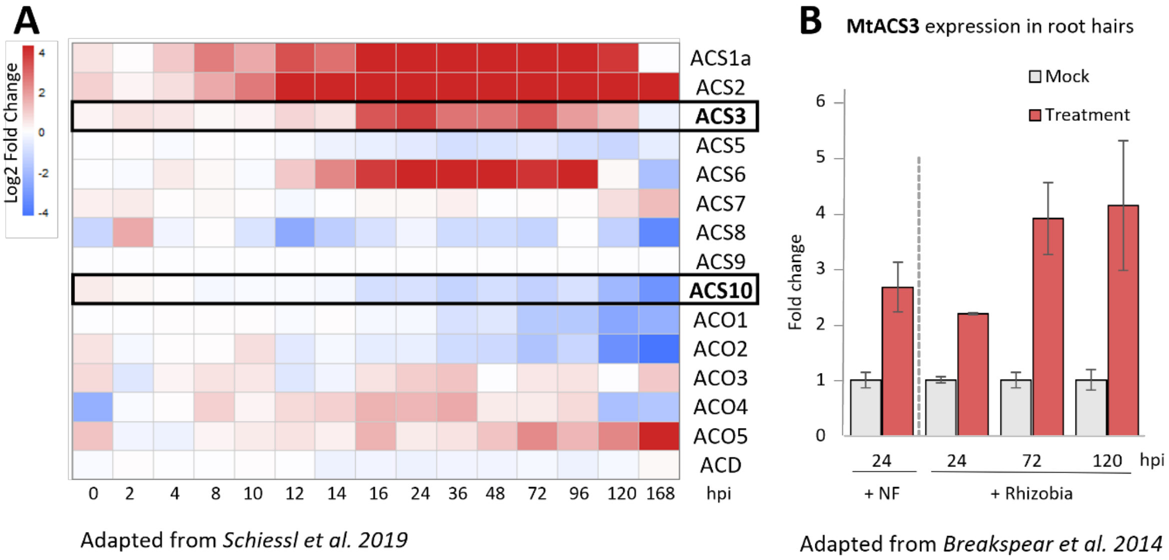

Supplemental Figure S2. Ethylene biosynthesis gene expression from published datasets. For reference, the gene names were unified according to Supplemental Table S1. (A) From Schiessl et al., (2019).(B) From Breakspear et al., (2014) Bars represent mean ± SE (n=3, independent biological replicates) (Supports Figure 2).

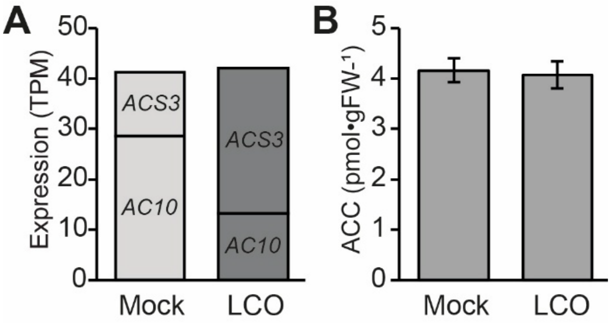

Supplemental Figure S3: The effect of 3 hours lipo-chitooligosaccharide (LCO) application on **(A)** cumulative *MtACS3* and *MtACS10* expression (TPM, transcripts per million), and **(B)** ACC levels. All bars represent means ± SE, (n=6); For B, no statistical significance differences (Student t-test, $P < 0.05$); n, 10-20 pooled root susceptible zones as independent biological replicates (Supports Figure 3).

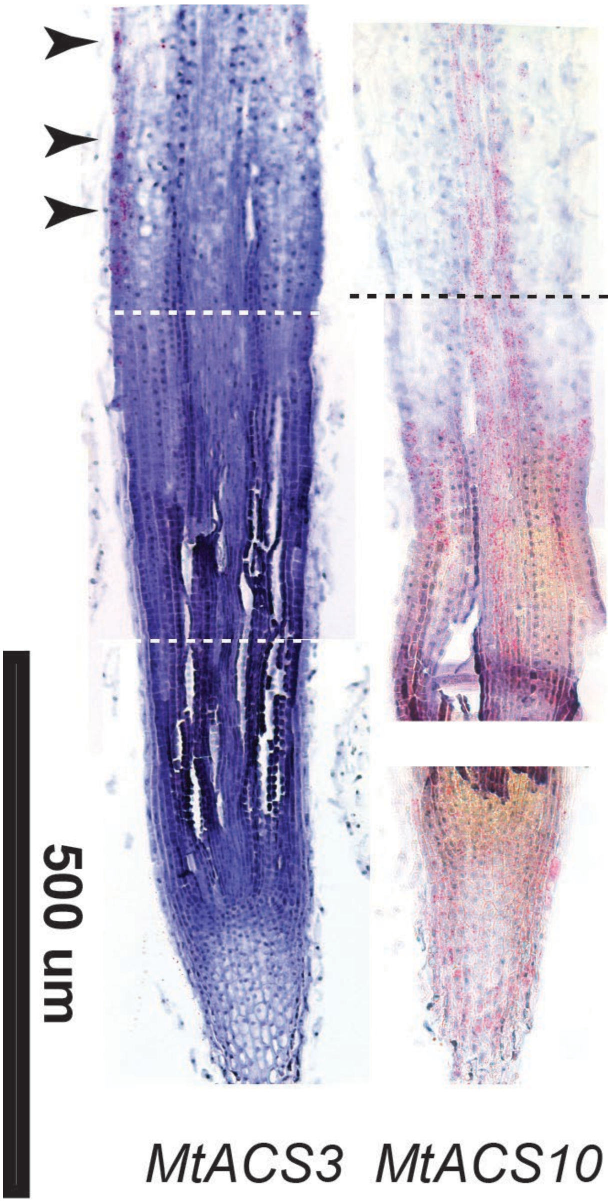

Supplemental Figure S4. The expression domain of *MtACS3* and *MtACS10* at the Medicago root tip. Composite image of a longitudinal sections of the untreated Medicago A17 wild-type root tip with *MtACS3* or *MtACS10* expression pattern visualized via RNA *in situ* hybridization. Hybridization signals appear as red dots (arrowheads highlight distinct expression domain of *MtACS3* in the epidermis and c1 layer of the start of the differentiation zone). Images from both roots are compiled from three consecutive images. Botted lines and white spacing mark the borders of these individual images (Supports Figure 3).

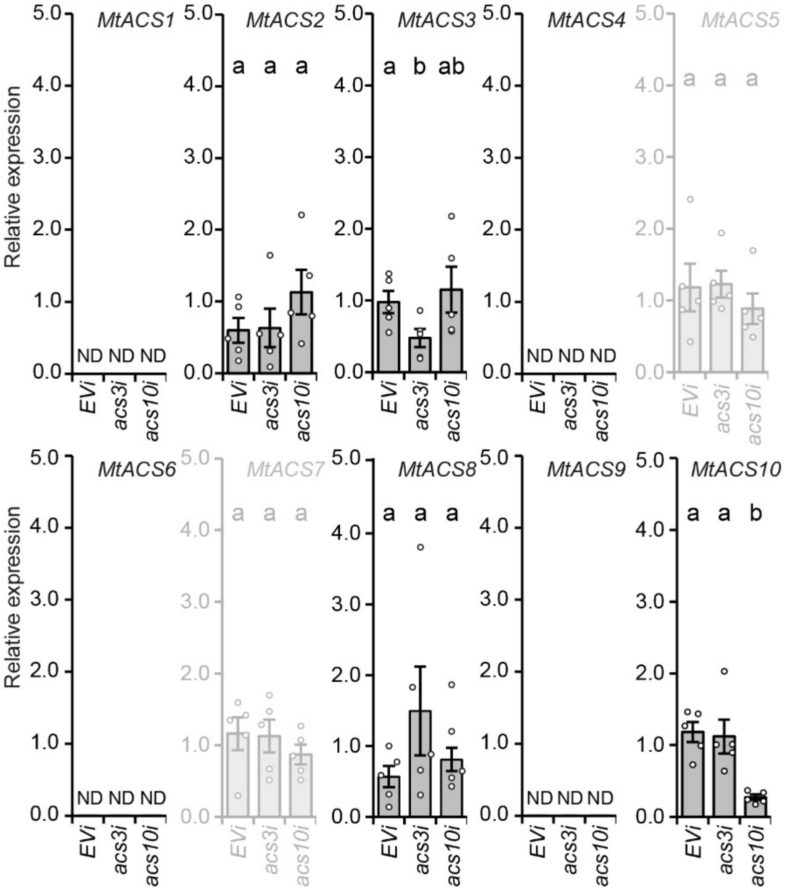

Supplemental Figure S5: Silencing and off-target effects of *ACS3*^{i} and *ACS10*^{i} on *MtACS1*, *MtACS2*, *MtACS3*, *MtACS4*, *MtACS5*, *MtACS6*, *MtACS7*, *MtACS8*, *MtACS9*, and *MtACS10* expression under mock conditions. Each dot represents an individual transgenic root; bars show mean ± SE Different letters indicate statistical significance between the Empty Vector (*EV*^{i}) and *ACS3*^{i} or *ACS10*^{i} line (n=5, one-way ANOVA followed by Tukey's HSD test, *P* < 0.05). Putative aminotransferases previously misidentified as ACC synthases in grey; ND, not detected; n, independent biological replicates (Supports Figure 4).

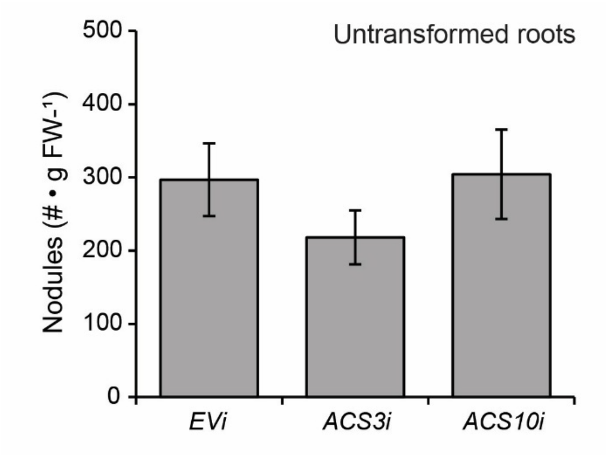

Supplemental Figure S6: Nodule number in non-transgenic roots of Empty Vector and ACS RNAi composite plants shows no significant differences. Numbers of nodules formed on non-transgenic roots belonging to the composite plants of the Empty Vector (*EV^i^*) control and the two RNA interference (*ACS3^i^* and *ACS10^i^*) lines (n>10). Bars represent means ± SE. Non-transgenic roots were harvested based on absence of *DsRed* expression. Bars represent means ± SE. Non-transgenic roots were harvested based on absence of *DsRed* expression. No statistical significance differences (one-way ANOVA followed by Tukey's HSD test, $P > 0.05$); n, non-transgenic roots from individual plants as independent biological replicates (Supports Figure 4).

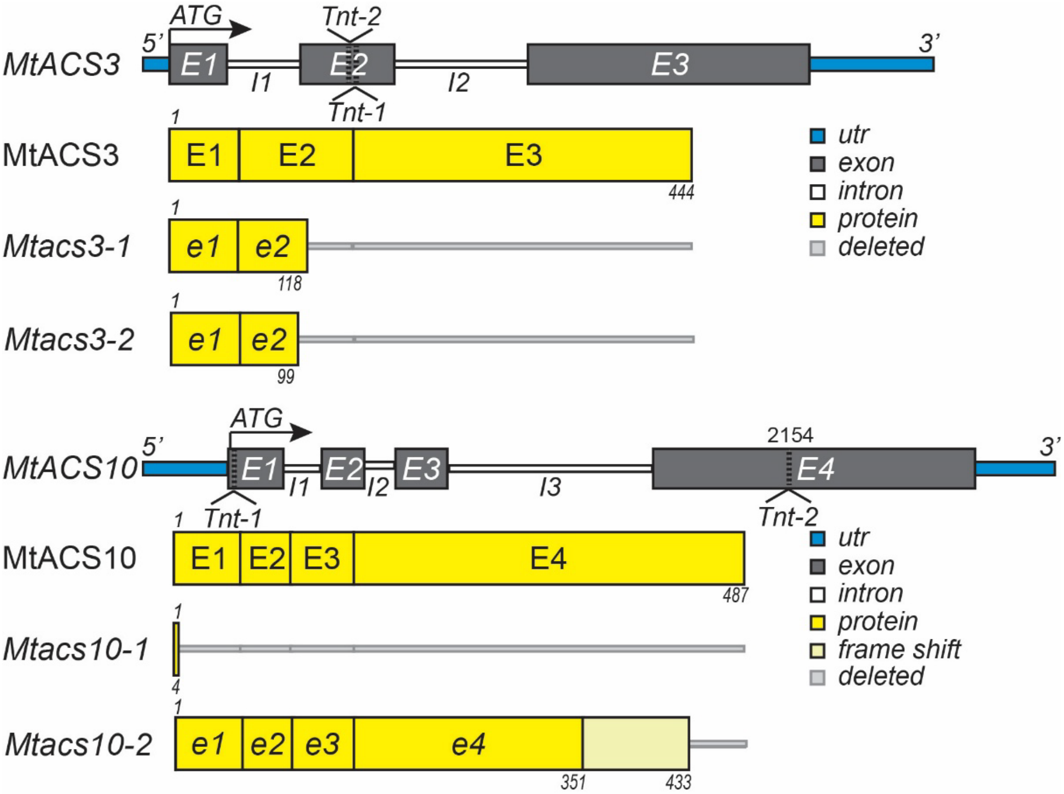

Supplemental Figure S7: *Tnt1* insertion mutations in *MtACS3* and *MtACS10*. Schematic representation of the gene structures of *MtACS3* (top) and *MtACS10* (bottom), showing untranslated regions (blue), exons (grey), introns (white), and predicted protein-coding regions (yellow). Positions of *Tnt1* retrotransposon insertions are indicated by triangles. The *Mtacs3-1* and *Mtacs3-2* allele carry an insertion in exon 2, resulting in a truncated predicted protein of 118 and 99 amino acids, respectively, compared to the full-length 444 aa MtACS3. The *Mtacs10-1* and *Mtacs10-2* alleles carry an insertion in either exon 1 and exon 4. The *Tnt1* insertion in *Mtacs10-1* leads to a severely truncated predicted protein of only 4 amino acids compared to the 487 aa full-length MtACS10, whereas *Mtacs10-2* leads to a frame shift at amino acid 351 followed by a premature stop codon at amino acid 433. Deleted regions are indicated in light grey (Supports Figure 4).

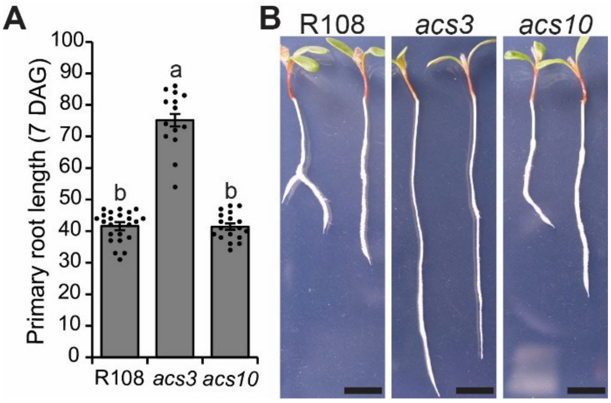

Supplemental Figure S8: Loss of *MtACS3*, but not *MtACS10*, increases primary root length in Medicago. **(A)** Quantification of primary root length at 7 days after germination (DAG) in wild-type R108, *Mtacs3*, and *Mtacs10* mutant seedlings (n>14). Each dot represents an individual plate grown root; bars show mean ± SE Different letters indicate statistical significance (one-way ANOVA followed by Tukey's HSD test, $P < 0.001$ for *Mtacs3* vs. R108 and *Mtacs3* vs. *Mtacs10*; n.s., not significant for *Mtacs10* vs. R108). **(B)** Representative seedlings of R108, *Mtacs3*, and *Mtacs10* at 7 DAG. Scale bars, 1 cm (Supports Figure 4).

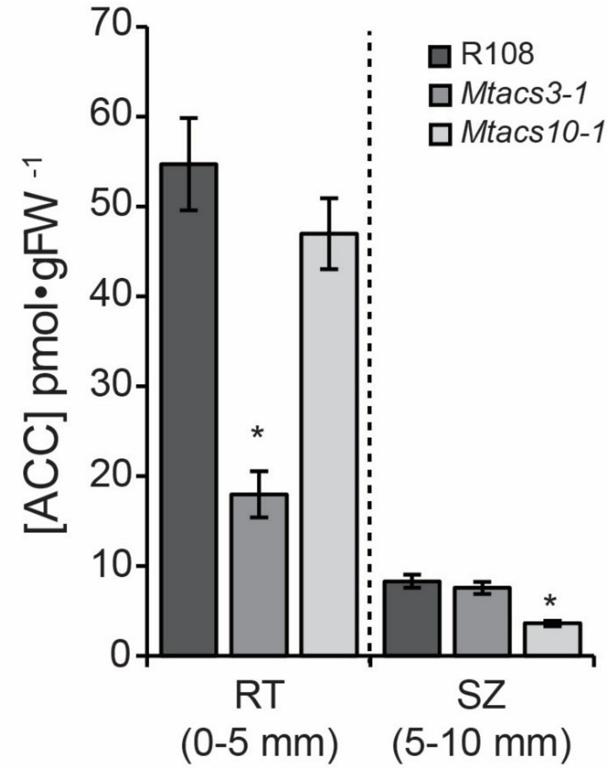

Supplemental Figure S9: ACC concentrations in wild type, *Mtacs3-1*, and *Mtacs10-1* roots of Medicago (R108) measured per gram fresh weight (FW) in the root tip (RT; 0–5 mm) and susceptible zone (SZ; 5–10 mm). Bars represent mean ± SE; n=5, independent biological replicates each consisting of ~24 pooled plate grown root tips separated into two 5 mm segments; different letters indicate statistical significance (paired Student t-test, $P < 0.05$) (Supports Figure 4).

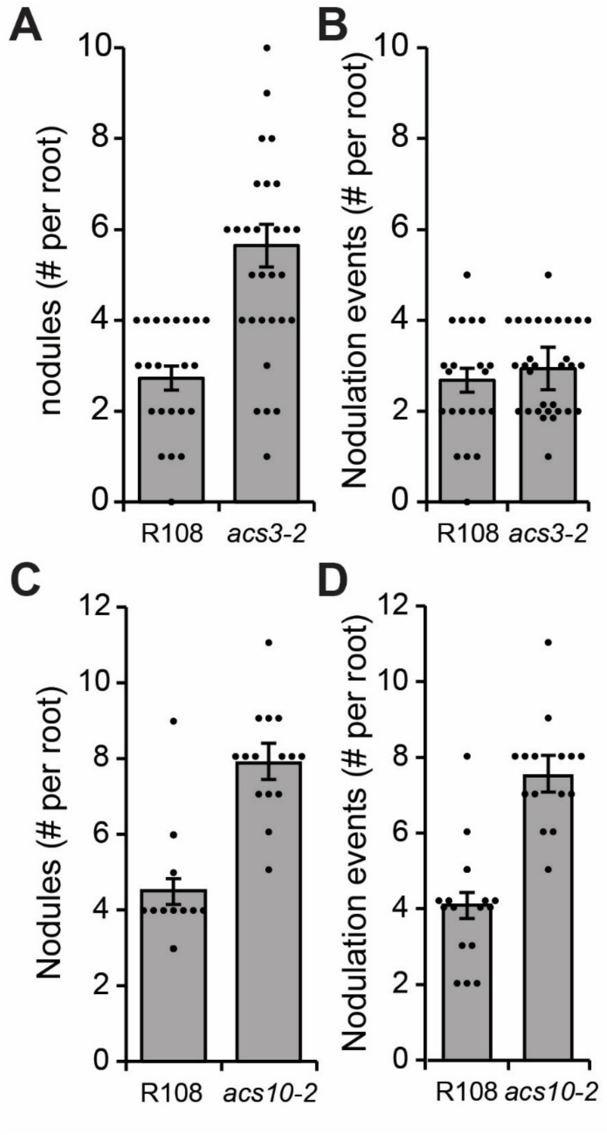

Supplemental Figure S10: Effect of *Mtacs3-2* and *Mtacs10-2* knockout mutations on nodulation in R108 plants grown on plates. (A) Average number of root nodules formed on R108 and *Mtacs3-2* mutants (n>22, independent roots of plate grown plants). (B) Number of nodule initiation sites per root (n>22, independent roots of plate grown plants, same plants as in A). (C) Average number of root nodules formed on R108 and *Mtacs10-1* (*acs10-2*) mutants (n>15, independent roots of plate grown plants). (D) Number of nodule initiation sites per root (n>15, independent roots of plate grown plants, same plants as in C). Each dot represents an individual root, bars represent mean ± SE; an asterisk (*) indicate significant differences (Student t-test, $P < 0.05$) (Supports Figure 4).

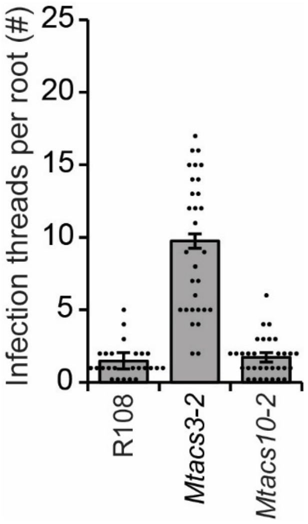

Supplemental Figure S11: Effect of *Mtacs3-2* and *Mtacs10-2* loss-of-function mutations on infection thread formation. Quantification of infection threads per spot-inoculation site following inoculation with GFP-labeled *Sm2011* (Sm2011-GFP) on wild-type R108, *Mtacs3-2*, and *Mtacs10-2*. Each dot represents an individual spot inoculated susceptible zone (n>24); bars represent mean ± SE; different letters indicate statistical significance (one-way ANOVA followed by Tukey's HSD test, $P < 0.05$) (Supports Figure 5).

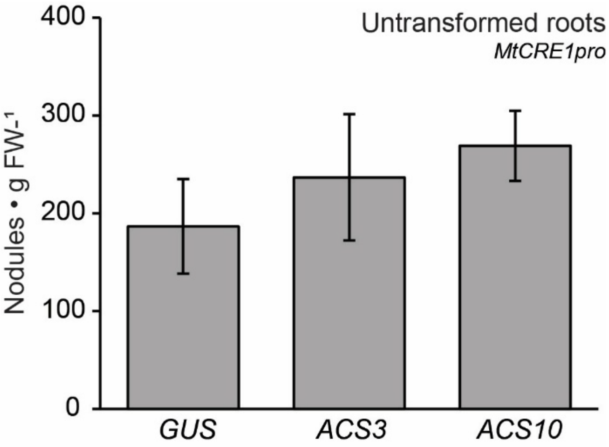

Supplemental Figure S12: Nodule number in non-transgenic roots of control *GUS* and ectopically induced *ACS* expression lines shows no significant differences. Numbers of nodules formed on non-transgenic roots belonging to the composite plants of the control *GUS* (*MtCRE1pro::GUS*) and two independent ectopically induced *ACS* expression lines, *ACS3* (*MtCRE1pro::ACS3)* and *ACS10* (*MtCRE1pro::ACS10*) (n>15). Bars represent means $\pm$ SE. Non-transgenic roots were harvested based on absence of *DsRed* expression. No statistical significance differences (one-way ANOVA followed by Tukey's HSD test, $P > 0.05$); n, non-transgenic roots from individual plants as independent biological replicates (Supports Figure 7).

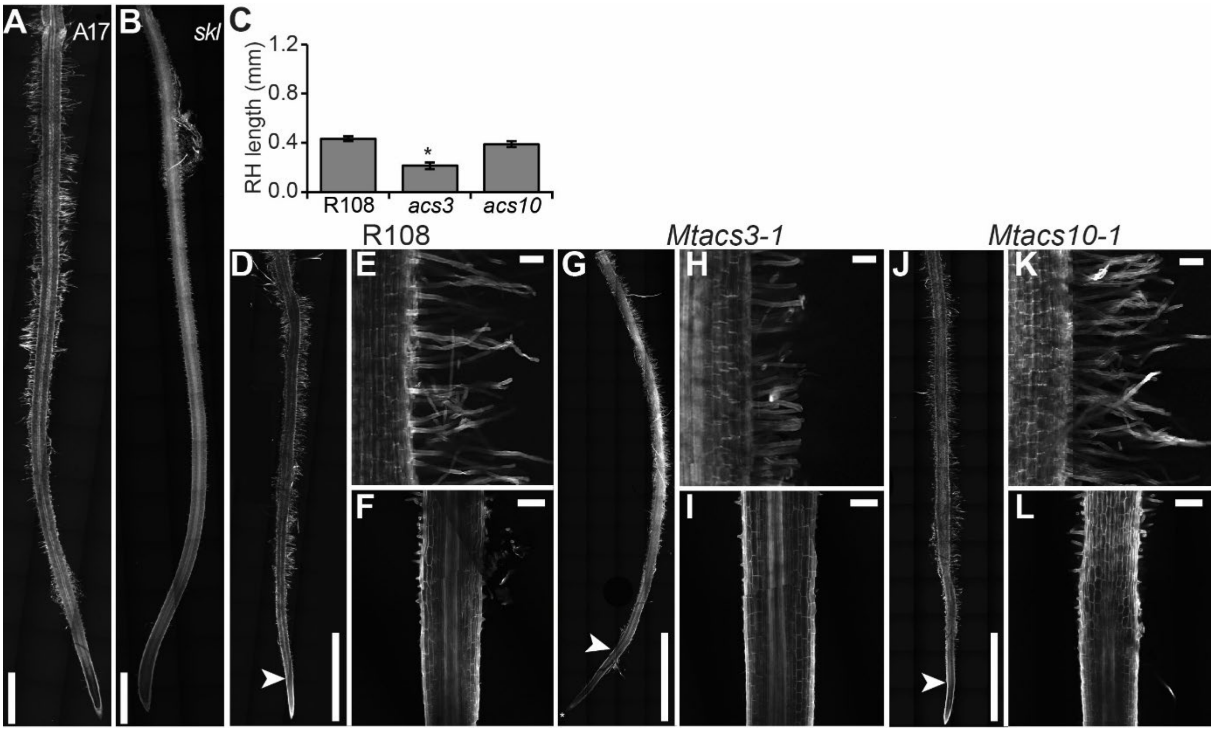

Supplemental Figure S13: Root hair development on wild-type Medicago A17, *skl* (*sickle*), wild-type Medicago R108, *Mtacs3-1*, and *Mtacs10-1*. **(A-B)** Full confocal images as used for cropped images in Figure 8G,H of the Medicago **(A)** wild-type A17 and **(B)** *skl* **(C)** Average length of the root hairs (RH) in the susceptible zone measured at roughly 4 mm distance from the root tip in wild-type R108, *Mtacs3-1*, and *Mtacs10-1.* Bars represent mean ± SE; n>6 (multiple root hairs from minimally 6 plants measured); an asterisk (*) indicate significant differences (paired Student t-test, $P < 0.05$). **(D-L)** Representative confocal images of the Medicago **(D-F)** wild-type R108, **(G-I)** *Mtacs3-1*, and **(J-L)** *Mtacs10-1* root tips and root hair zones. **(D, G, J)** Confocal tile image of the Medicago **(D)** wild-type R108, **(G)** *Mtacs3-1*, and **(J)** *Mtacs10-1* root (Arrowhead points at first root hair, asterisk (*) in G marks root tip, scale bars 5 mm). **(E, H, K)** Confocal image of root hairs in the root susceptible zone of Medicago **(E)** wild-type R108, **(H)** *Mtacs3-1*, and **(K)** *Mtacs10-1* (scale bars 100 µm). **(F, I, L)** Confocal zoom in image of first root hairs of Medicago **(F)** wild-type R108, **(I)** *Mtacs3-1*, and **(L)** *Mtacs10-1* (scale bars 200 µm) **(A, B, D, G, J)** Images were generated using the Zeiss 710 tile scan option (Zen 2009 software, default settings) (Supports Figure 8).

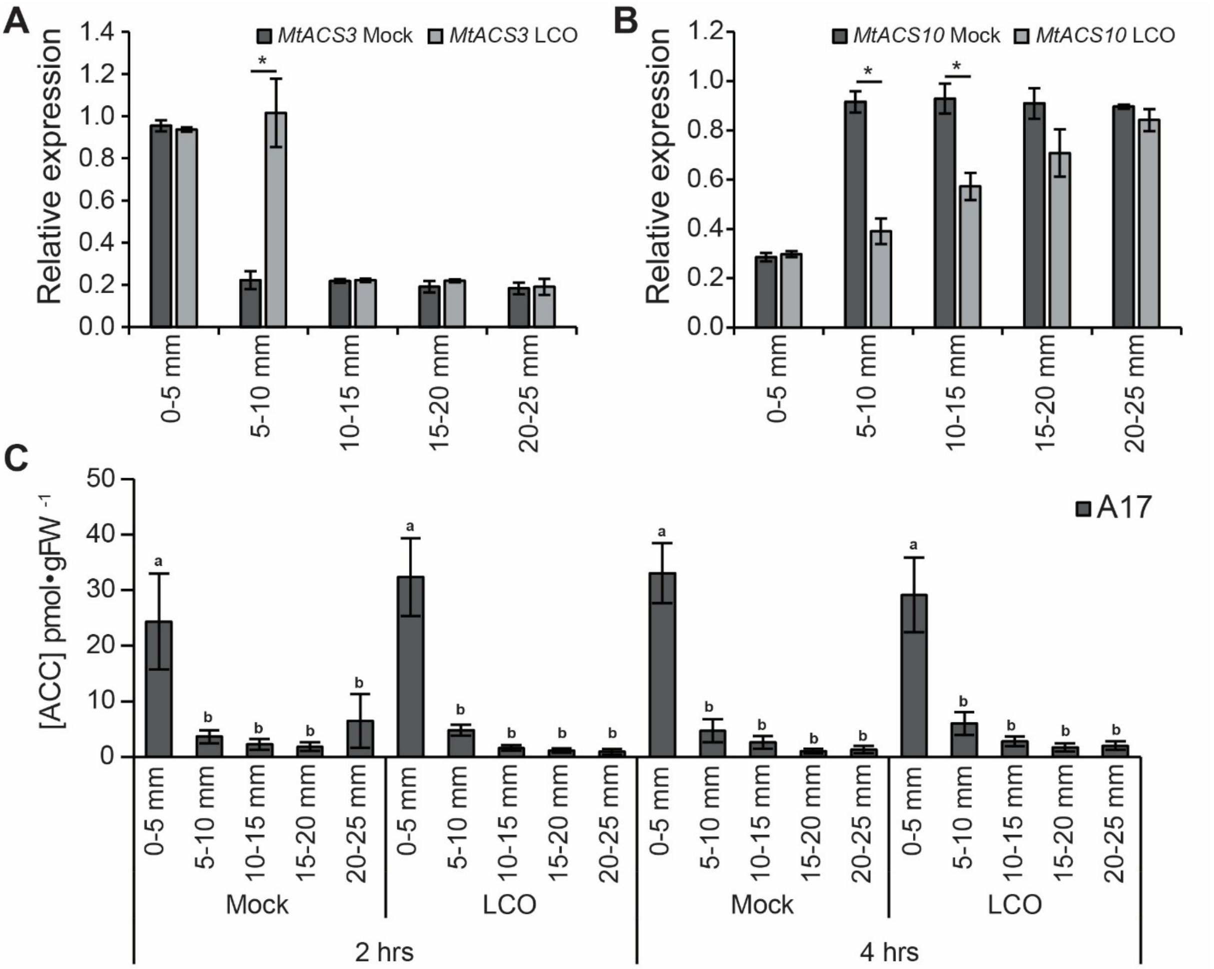

Supplemental Figure S14: *MtACS3* and *MtACS10* expression dynamics and ACC concentrations along the Medicago wild-type Jemalong A17 root axis and R108 mutants. (A) Relative expression over the root axis per 5 mm zones under Mock and lipo-chitooligosaccharide (LCO) application in Medicago A17 of (A) *MtACS3* and (B) *MtACS10*. Bars represent mean ± SE; n=3, independent biological replicates each consisting of the susceptible zones of ~16 pooled plate grown plants. (C) Concentrations of ACC along the root axis of Medicago A17 per gram fresh weight (FW) in response to Mock or LCO treatment (2 and 4 hours). Bars represent mean ± SE; n=5, independent biological replicates each consisting of ~40 pooled mass inoculation plate grown root tips separated into 5 segments (0-5 mm, 5-10 mm, 10-15 mm, 15-20 mm and 20-25 mm measured from the root apex); different letters indicate statistical significance (two-way ANOVA followed by Tukey's HSD test, $P < 0.05$) (Supports Figure 8).

Supplementary Table S1: Overview of ethylene biosynthesis genes with version ID and common name used in different publications. Based on Gómez-Fernández et al., (2025). "-" gene absent from the dataset, "nn" gene was not named with a number.

Common Gene Name	A17 (MtrunA17r5.0-ANR)	A17 (Mt4.0v1)	Breakspear et al. 2014	Larrainzar et al. 2015	Van Zeijl et al. 2015	Schiessl et al. 2019
ACS1a	MtrunA17_Chr8g0389311	Medtr8g101750	-	-	**ACS1**	*nn*
ACS1b	MtrunA17_Chr8g0389321	Medtr8g101820	-	**ACS7**	-	-
ACS2	MtrunA17_Chr4g0054371	Medtr4g097540	-	**ACS4** contig_12436_1	**ACS2**	*nn*
**ACS3**	MtrunA17_Chr6g0488041	Medtr6g091760	Mtr.20234.1.S1_at	*nn*	**ACS3**	*nn*
ACS4	MtrunA17_Chr3g0135251	Medtr3g103550	-	-	-	-
ACS5	MtrunA17_Chr5g0398431	Medtr5g011400	-	-	-	*nn*
ACS6	MtrunA17_Chr5g0400831	Medtr5g015020	-	**ACS8**	-	*nn*
ACS7	MtrunA17_Chr6g0475681	Medtr6g463260	-	-	-	*nn*
ACS8	MtrunA17_Chr7g0248681	Medtr7g079080	-	*nn*	-	*nn*
ACS9	MtrunA17_Chr8g0348351	Medtr8g028600	-	-	-	*nn*
**ACS10**	MtrunA17_Chr8g0387481	Medtr8g098930	-	contig_49261_1	-	*nn*
ACO1	MtrunA17_Chr2g0289341	Medtr2g025120	-	*nn*	-	*nn*
ACO2	MtrunA17_Chr3g0121141	Medtr3g083370	-	*nn*	-	*nn*
ACO3	MtrunA17_Chr3g0125201	Medtr3g088565	-	-	-	*nn*
ACO4	MtrunA17_Chr5g0439011	Medtr5g085330	-	*nn*	-	*nn*
ACO5	MtrunA17_Chr6g0488511	Medtr6g092620	-	-	-	*nn*
ACD	MtrunA17_Chr8g0393311	Medtr8g107670	-	-	-	*nn*

Supplementary Table S2: Primers and Gene IDs (Medicago genome v4.01) used in this study.

Primer name	Sequence	Gene ID Mt4
MtACO1_Fw	ATTGGAGAAACTGGCAGAGG	Medtr2g025120
MtACO1_Rv	CCAGCATCTGTGTGTGCTCT	
MtACO2_Fw	CAAAGGGACCAACTTTTGGA	Medtr3g083370
MtACO2_Rv	GGGAGGGACATCTACCCAGT	
MtACO3_Fw	CCGGAACTTGTGAATGGTCT	Medtr3g088565
MtACO3_Rv	ATCTGCCATTGCTCAGGACT	
MtACO4_Fw	CATCGTTGTCAACCTTGCTG	Medtr5g085330
MtACO4_Rv	GGAGCAGGGTAAATGACAGC	
MtACO5_Fw	GATGCTGGTGGAATCATCCT	Medtr6g092620
MtACO5_Rv	TCCATTCTTGTCAGGCATCA	
MtACS1_Fw	CGGAGATGCTTTGCTTGTTCC	Medtr8g101750
MtACS1_Rv	CAGCACTCCACTCACTTTCATG	
MtACS2_Fw	TGGTTTCGTGTGTGTTTCGC	Medtr4g097540
MtACS2_Rv	TTGCCAGTGGACTCAACAAC	
MtACS3_Fw	TGGGCTTGGCAGAAAATCAAG	Medtr6g091760
MtACS3_Rv	ACCACCTTTTCCCCATGTTG	
MtACS4_Fw	TGCAACTTTTGAGGCAGAGA	Medtr3g103550
MtACS4_Rv	AATCCTTCGCATTGCAACTT	
MtACS5_Fw	CTGCTGGGCTGACATGAGTA	Medtr5g011400
MtACS5_Rv	CCCGATCCGTTCCACTACTA	
MtACS6_Fw	TCATGTTTTGCCTTGCTGAA	Medtr5g015020
MtACS6_Rv	AAGCCGATTCTGTGATTTGG	
MtACS7_Fw	CAACGGTTTTCTCCTGGGTA	Medtr6g463260
MtACS7_Rv	CGAAATAGAGCCACGAGAGG	
MtACS8_Fw	GGGTCTTCCGGGTTTTAGAG	Medtr7g079080
MtACS8_Rv	AAAATTCACGTCGCTCTGGT	
MtACS9_Fw	ATCCAGGATTCGTTCGTGAC	Medtr8g028600
MtACS9_Rv	CTAATGGGTTGGAGGGGTTT	
MtACS10_Fw	CGAACTCGTCATCAGACAGC	Medtr8g098930
MtACS10_Rv	TGACCGTAACCTCGTTCACA	
MtACD_Fw	AAACAAAGTGCGGAAATTGG	Medtr8g107670
MtACD_Rv	GGGATCTTGGTCAACGAGAA	
MtNIN_Fw	GGGAGAAAGTCCGGGGACAA	Medtr5g099060
MtNIN_Rv	GACACACACCGATGCTCTTTGC	
MtACT_Fw	GCAAAGGCAGAATATGATGAAT	Medtr2g008050
MtACT_Rv	CCACTATGACTGCCAGAACACTTA	
MtPTB_Fw	TGAACCAGTGCCTGGAATCCT	Medtr3g090960
MtPTB_Rv	CGCCTTGTCAGCATTGATGTC	
MtUBQ_Fw	CACCTCCAATGTAATGGTCTTTCC	Medtr4g091580
MtUBQ_Rv	CCCTTCATCTTGTCCTTCGTCTG	
MtACS3_RNAi_Fw	CACCCCCTACTTTGCTGGATGGAA	Medtr3g088565
MtACS3_RNAi_Rv	CAACTGCGGCTAATGAGCTT	
ACS10_RNAi_Fw	CACCAACCACCCCATACTTCATTCC	Medtr8g098930
ACS10_RNAi_Rv	CCAACCAAAAATCCTCAAGG	

Supplementary Table S3: Gene probe sets used for ViewRNA *in situ* hybridization

Gene name	Gene ID Mt4 / r5.0	Assay ID according to ThermoFisher Scientific
MtACS3	Medtr6g091760 / MtrunA17_Chr6g0488041	catalogue number VF1-6000770
MtACS10	Medtr8g098930 / MtrunA17_Chr8g0387481	catalogue number VF1-6000771

Supplementary Table S4: Sequences use for RNAi constructs.

MtACS3^{i} _fragment

^{CACC}CCCTACTTTGCTGGATGGAAAGCATATGATGAAAACCCTTATCATGAATTAACTAACTCTTCTGGTGTTATA
CAAATGGGATTGGCAGAAAATCAAGTTTCATTTGATTTGGTAGAAAAGTATTTGGAAGTGCACCCGGAAGATTAC
AATGGTTTCAGAGAAAATGCATTATTTCAAGACTATCATGGACTTAAATCATTCAGAACTGCAATGGCAAGTTTC
ATGGAACAAATAAGAGGTGGTAAAGCTACATTTGATTCGGAAAGAATAGTCATCACTGCCGGAGCAACTGCGGCT
AATGAGCTT

Note: CACC added for TOPO cloning

MtACS10^{i}_fragment

^{CACC}AACCACCCCATACTTCATTCCTACACATTGCAATCTCTTCTAACTTATTCTTACACCTTCAAACATAAGAAG
AATTCCCACACAAATTCGCTTCTTCCTCGTACTTTTTTGTTTTTGTTTTTGAATCTCATTGATTTCATCACATTT
TCTATTAAATTAATTAATATTTTAGACTATAGTAATTAATAATGGGACTTGTGAGCATGGACCAACCTCAATTGT
TGTCCAAGATAGCCACTGGTGATGGACATGGTGAAACATCATCTTACTTTGATGGATGGAAAGCTTATGATAAAA
ACCCTTTTCATCCAACCAAAAATCCTCAAGG

Note: CACC added for TOPO cloning

Supplemental table S5. Sequences used for ectopic expression of *MtACS3* and *MtACS10*.
EC74831; *MtCRE1p-MtACS3-t35S*
EC74832; *MtCRE1p-MtACS10-t35S*
EC74833; *MtCRE1p-GUS-t35S*
EC74963; *MtACS3p-GUS-ACS3-3`UTR*
EC74964; *MtACS10p-GUS-ACS10-3`UTR*

MtACS3
ATGGGTCTTGAGATTGAACAAGAACACCCTTGTGTTGAACTTTCAAATATTGCAACTTCTGAAACTCATGGAGAA
AATTCTCCATACTTTGCTGGATGGAAAGCCTATGATGAAAACCCTTATCATGAAATAACTAACCCTTCTGGAGTT
ATACAAATGGGCTTGGCAGAAAATCAAGTATCATTTGATTTACTTGAAAAATACTTGGAAGAACACTCAGAGGCT
TCAACATGGGGAAAAGGTGGTTCAAGTTTTAGAGATAATGCATTATTTCAAGACTATCATGGACTTAAATCATTC
AGAAAAGCAATGGCAAGTTTCATGGAAAAAATTAGAGGAAATAAAGCAAAATTTGATTATGAAAGAATCGTCCTC
ACTGCTGGTGCTACTGCTGCCAATGAGCTCTTGACTTTCATTCTTGCAAATCCAGGAGATGCTTTACTTGTTCCA
ACACCATACTATCCTGGATTTGATAGAGATTTGAGATGGAGAACTGGTGTAAACATAATTCCAATCCATTGTGAT
AGCTCAAACAATTTTCAAATCACACTTGAAGCATTAGAAACTGCATACAAAAATGCAGAATCCATGAACATGAAA
GTAAAAGCAGTACTTATAACCAACCCATCAAATCCATTAGGCATATCGATTCAACGTTCAGTTCTCGAGGACATT
CTGAACTTCGTGACTCGCAAGAACATACACCTTGTCTCAGACGAAATCTACTCGGGCTCAGTTTTCTCTTCACAT
GAATTCATAAGCATAGCCGAGATTCTTGAATCTCGTCAATACAAAGACGCGGAAAGATGTCACATTGTTTATAGT
CTTTCTAAAGATCTCGGTCTACCAGGTTTCAGAGTCGGAACAATTTATTCCTACAACGATAAAGTTGTTACAACA
GCACGAAGAATGTCGAGTTTTACCTTAATATCTTCACAAACACAACATCTTTTAGCATCAATGTTGTCAGATGAA
AGTTTCACTGATAATTACATCAAGGTCAATAGAGAAAGATTAAGGAAAAGATATGAAATGATCATTGAAGGTTTG
AAAAGTGCTGGAATTGAATGCTTGAAAGGTAATGCAGGGTTGTTTTGTTGGATGAATATGAGTCCAATGTTGGAA
AGTAATACAAGAGAAGGTGAATTGAAGCTTTGGAATGAGATTTTGAATGAAGTTAAGCTTAATATTTCACCAGGG
TGTTCTTGTCATTGTTCCGAACCCGGTTGGTTTAGGGTTTGTTTTGCAAATATGAGTGAAGAAACACTTGAACTT
GCACTCAAAAGAATACGTGATTTCATGAATAACAAGGACAGAAAGGATATAGGAATATAA

MtACS10
ATGGGACTTGTGAGCATGGACCAACCTCAATTGTTGTCCAAGATAGCCACTGGTGATGGACATGGTGAAACATCA
TCTTACTTTGATGGATGGAAAGCTTATGATAAAAACCCTTTTCATCCAACCAAAAATCCTCAAGGTGTTATCCAA
ATGGGTCTTGCAGAGAATCAGCTTACTGCTGATTTGGTTCAAAATTGGATAATGAGTAACCCAGAAGCCTCAATT
TGTACTCTAGAAGGAGTACACAATTTCAAAGAAATGGCTAATTTTCAGGATTATCATGGTCTACCAGAGTTCAGA
AATGCTGTGGCTAAATTCATGTCAAGAACAAGAGGAAATAGAGTGACATTTGATCCTGATCGTATTGTCATGAGT
GGTGGAGCAACTGGAGCACATGAGGCCACTGCCTTTTGTTTGGCAGATCCTGGTGATGCTTTTTTGGTGCCTACA
CCTTACTATCCAGGATTTGATCGAGATTTGAGGTGGAGAACAGGGGTTAAACTTGTTCCAGTTATCTGCGAAAGT
GCAAACAATTTCAAATTAACAAAACAAGCCTTAGAAGAAGCATATGAAAAAGCCACAGAAGATAACATCAGAATT
AAAGGTTTACTCATAACAAATCCCTCAAATCCATTAGGCACAGTTATGGACAGAAACACATTAAGAACCGTTGTA
AATTTCATCAACGAAAAGCGTATTCACTTAATAAGCGATGAAATTTACGCTGCAACGGTTTTTAGCCACCCAAGT
TTCATAAGCATAGCTGAAATATTAGAACATGACACAGACATTGAATGTGACCGTAACCTCGTTCACATAGTTTAC
AGTCTTTCAAAAGACATGGGATTCCCTGGTTTTAGAGTTGGTATAATATACTCTTATAATGATACCGTTGTAAAT
TGTGCACGAAAAATGTCAAGTTTTGGATTAGTTTCAACACAGACACAATACTTGATGGCGAAAATGCTGTCTGAT
GACGAGTTCGTTAAAAAGTTTCTTACTGAAAGTGCAAAGAGGTTAGCACAAAGGTACAGAATTTTCACCAGTGGA
TTAACCAAAGTTGGAATTAATTGTTTACAAAGTAACGGTGGACTTTTTGTGTGGATGGATTTGAGAGGACTTCTT
AAGGAAGCTACATTTGAATCAGAATTGGAACTATGGAGAGTGATTATTCACGAAGTTAAGATTAATGTTTCACCT
GGAGTTTCTTTTCATTGTTCTGAGCCAGGGTGGTTTAGAGTGTGTTATGCTAACATGGATGATAGAGATGTGCAA
ATTGCTTTACAAAGGATTAGGTCATTTGTGGTTCAGAATAATAAGGAGGTTATGGTGTCTGAGAAGAACACTAAA
CCTTGTTGGCATAGTAATTTGAGGTTAAGCCTTAAAACAAGAAGGTTTGATGATATTGTAATGTCACCTCATTCT
CCATTTCCTCAGTCACCTCTTGTTAAAGCCACTACTTGA

GUS (pICH75111)
ATGGGTCAGTCCCTTATGTTACGTCCTGTAGAAACCCCAACCCGTGAAATCAAAAAACTCGACGGCCTGTGGGCA
TTCAGTCTGGATCGCGAAAACTGTGGAATTGATCAGCGTTGGTGGGAAAGCGCGTTACAAGAAAGCCGGGCAATT
GCTGTGCCAGGCAGTTTTAACGATCAGTTCGCCGATGCAGATATTCGTAATTATGCGGGCAACGTCTGGTATCAG
CGCGAAGTCTTTATACCGAAAGGTAAGTCTTACTCTCTCTTTTTTGGTCTGTATTTTTAATTTTTTGAAGTATAC
TATTTGTACTGACGCTAATAATCTTTTTTCAGGTTGGGCAGGCCAGCGTATCGTGCTGCGTTTCGATGCGGTCAC
TCATTACGGCAAAGTGTGGGTCAATAATCAGGAAGTGATGGAGCATCAGGGCGGCTATACGCCATTTGAAGCCGA
TGTCACGCCGTATGTTATTGCCGGGAAAAGTGTACGTATCACCGTTTGTGTGAACAACGAACTGAACTGGCAGAC
TATCCCGCCGGGAATGGTGATTACCGACGAAAACGGCAAGAAAAAGCAGTCTTACTTCCATGATTTCTTTAACTA
TGCCGGAATCCATCGCAGCGTAATGCTCTACACCACGCCGAACACCTGGGTGGACGATATCACCGTGGTGACGCA
TGTCGCGCAAGACTGTAACCACGCGTCTGTTGACTGGCAGGTACTTCATGCTTCAACGTGTAACTTAAGAGATAC
TGTGTGAAATTTTATATTTCCATACATTTGCTTGACCTTTGCTTTTTGTCAATTTTTTTCCCCTTACAGGTGGTG

GCCAATGGTGATGTCAGCGTTGAACTGCGTGATGCGGATCAACAGGTGGTTGCAACTGGACAAGGCACTAGCGGG
ACTTTGCAAGTGGTGAATCCGCACCTCTGGCAACCGGGTGAAGGTTATCTCTATGAACTGTGCGTCACAGCCAAA
AGCCAGACAGAGTGTGATATCTACCCGCTTCGCGTCGGCATCCGGTCAGTGGCAGTGAAGGGCGAACAGTTCCTG
ATTAACCACAAACCGTTCTACTTTACTGGCTTTGGTCGTCATGAAGATGCGGACTTGCGTGGCAAAGGATTCGAT
AACGTGCTGATGGTGCACGACCACGCATTAATGGACTGGATTGGGGCCAACTCCTACCGTACCTCGCATTACCCT
TACGCTGAAGAGATGCTCGACTGGGCAGATGAACATGGCATCGTGGTGATTGATGAAACTGCTGCTGTCGGCTTT
AACCTCTCTTTAGGCATTGGTTTCGAAGCGGGCAACAAGCCGAAAGAACTGTACAGCGAAGAGGCAGTCAACGGG
GAAACTCAGCAAGCGCACTTACAGGCGATTAAAGAGCTGATAGCGCGTGACAAAAACCACCCAAGCGTGGTGATG
TGGAGTATTGCCAACGAACCGGATACCCGTCCGCAAGGTGCACGGGAATATTTCGCGCCACTGGCGGAAGCAACG
CGTAAACTCGACCCGACGCGTCCGATCACCTGCGTCAATGTAATGTTCTGCGACGCTCACACCGATACCATCAGC
GATCTCTTTGATGTGCTGTGCCTGAACCGTTATTACGGATGGTATGTCCAAAGCGGCGATTTGGAAACGGCAGAG
AAGGTACTGGAAAAAGAACTTCTGGCCTGGCAGGAGAAACTGCATCAGCCGATTATCATCACCGAATACGGCGTG
GATACGTTAGCCGGGCTGCACTCAATGTACACCGACATGTGGAGTGAAGAGTATCAGTGTGCATGGCTGGATATG
TATCACCGCGTCTTTGATCGCGTCAGCGCCGTCGTCGGTGAACAGGTATGGAATTTCGCCGATTTTGCGACCTCG
CAAGGCATATTGCGCGTTGGCGGTAACAAGAAAGGGATCTTCACTCGCGACCGCAAACCGAAGTCGGCGGCTTTT
CTGCTGCAAAAACGCTGGACTGGCATGAACTTCGGTGAAAAACCGCAGCAGGGAGGCAAACAATGA

MtCRE1$_{pro}$
GGAGCCTAGAACCAATATAAAGACTATTTTTATTGTCAAAATCTAGACTTATTAGAACTTTTTTGTTTTCTAATT
TCCAAATGAGTTATATTATATGAACAATTTTTTCTTGTGACAACTTGATTGACAATCAAATTATACAAAGAAATT
TAGATAAATATACAAAAATCAAAGCAATAGAGAGAGAAAGTAGATAAATAATGTGAGTATGAGAGATAAAATTGT
CACAAAAGTTGTCAAAAATGATTGTTCAATTATCATTTTTCTTTTCAAATATTGAACTTTTTCTTACAAGATACA
AATGAGGCATTCTTCGATAGTCACGAACACAAATGCATAGTGTTTGGACACACACATATAATTTATATGTCATTT
CTTGAAATACTAAATTATTTTATTATCAATTTCTTTTTCATTATTAAAAAAAATTGTAATGTGTACGCGCCTAAA
CACTATCCATTTGTATTTGTGATTTCTCGATGCCAAGACTAAACTTGATGGCAATTATTTTATTGAAAAATTTGA
TAGCAATTAGTACCACTTAAGAATTAAATTGGTGGTATATTCATCTATTCTTTCTTTAGAGATGTAATCATGCCT
TTATTTGAATAAAGTGCCACAACGAGATAAGGTCACCTTAATAATTTGGAAGAGGACATTCTATCTTTCTTTTTT
TAACATTACATTTATTATTTTTAATGAAGTCGCAAAGATGTAAAAAAATTGTCCAAACAACAAAGTAGGGAACCA
ATCAAACCGTTTTTTCTTTTTCGTTGCAAAGTTAAGCTAAATGAAATGAGTAGCAATATTTGAATAACCATGTTG
GATAACTTATGTGATAATTTTTTTTCTCTCTTTTTATTGGTCAAAATCAATGGAAAGAGAAAAATAAGAGAGAGA
AAAAAAGTATAATATGAGTATGAGAGAGAAATTTGTAAAAAAAAAAAAATCATAAAATGGTTATACAAATGTAATT
TATCAAAAGAAATAACTAAATTAACTGTTTACCAACAATGAATGTATGAGTACTACTATACATAGATTATTAAGG
ATACATTAGTCACAACATTAATTAATACAAAACAACAAAGTTAATTAATGTTGATTATCCATTGAGGGTAGTTAT
GGAACTCTAAATAATAAAAAGGGTGGGTGTTTAGGGTAATTCACATGGGAATAAGATCCTAGCATTGTCGCAAGT
CACTATCTTTCAGATTTGTGATTGTGATTTTTTCTATCTTTCTCTCTCCTCTGTTCCACTACACAACATTGTTTA
ACGTTGGAACACATAGAAATAGTGAGAAAGACCCATTTGAAGATTCAACTCTAGAATGTGAAAAAGTTTCAATTC
TTACATTCATTTTCCAAAGTTAGTAATAATAGCTTAACTGGGTCAGTTCATTCTCCATTGAAGCTTCTTTTCAAA
GTGCTAGTTGAAAAAAGATGCTAACTTGAGGAGAAAATATTGTTGGGTTGAAAGAGAGAGTTTTGACTTGACTAG
AGTGTGAAGTAGAAGGGAGACAACAACAGCGTATCAGAGAAAATCGAAAAAAATTCGATTTAGTTTTAGTAATGT
CCTGTTTTGTGCTTAAAAAATAAAGAAAGTAAAGTAAGAAAGCATATATAAAAAGGTTGTATAAAAAACAAAAAC
TAGTACAGAAAGAGAAAGGTGCAGTGCAGTGCAGTGCAGATCAAGACGAACCCAACCAACCAATCTGCTCTCAAC
TGTGTTCATTGTATTGTTTCTCTTTCCTATTCCTATACAGGTAAACATTGTTTCTCTAACTTACTCATTGGGTTC
TGTGTGTGCTTCGCTGTTTTTTTTTTTTTTTTTTTGTTTAGTATGGTTTGTGTGTTTTATTTTTGAGGTCAATGTT
TGGTTTTTTTGATTAAACAAATAAAAGTGTGAACGTGCTTTTGTTTGTCCTTGAGCAGAGACTGGTTTCACGCCT
AGAGAGCATGATTAGAAGTCTCTTGAATTTTTAATTCATAATATTGGCTGTTGAGAGAAAGGAGAAGGTGCTTCT
TTGGAATTTGAGCTGTTTTTCCATCTTTTGAGAGCCATGCTTGTCTCTATTTCCCTTCTTTTTAATTCTTTCTCT
CTTTTCTCTCACTTCTTTTTTTTTATTCTCTAATTTTCTGTGTAATTTTTCAAATGAATTTTCCTCAATCATTCAT
CATGCTTGGTTTTGCTTTTTTTTATTTGATTCTTGTTTCCTAAGAGGCTTTGTTTTGGTTTGGTTTGAATCACGCA
AAACAATGCTGTAATGATGCTGTACTTGGTTTTTTGTTCTGTGAACTTTTTCTTTCAACCATCAGATAAAAGTGT
CTGAGATTTTAGGACTACTTTACACTTTAAGCAAAGTAAAAGCTACTTCCTATATATAAAAAAGTTTGTGCTCTG
TTAATTGAGTATCTTTTGGGACTTCCATAGATATGAAGAAGAGTTCAGAGAAATAGGCAGAAAGAAAGAGATTTG
GTGTTAGTTGGTGTTGTAAATG

t35S (pICH41414)
CTTCTCTAGCTAGAGTCGATCGACAAGCTCGAGTTTCTCCATAATAATGTGTGAGTAGTTCCCAGATAAGGGAAT
TAGGGTTCCTATAGGGTTTCGCTCATGTGTTGAGCATATAAGAAACCCTTAGTATGTATTTGTATTTGTAAAATA
CTTCTATCAATAAAATTTCTAATTCCTAAAACCAAAATCCAGTACTAAAATCCAGAT

MtACS3p

AACCCCCTAATTTCCTAACAATAACAAATTGTTTGGTTCAGTTTTAACGTTAAAAATATGAATTGGACACTCAAC
TCAAACCAAAGACAATTGATCAGATTCAGTAATGAGTTTGGTCAAATTAGCCCAACCCAACCTCCAAACACTCCT
AATCAGAGGTGGATAAAGTGGTGGTCGTCAATGGTGGGTGGTGGTTAGGGTTTACTGGGGGTAACATGAGTGGTG
GTAGAAGGTGGTCAAAATAATGGTCAGATAGGTGATAAGAGTTGGCCAACAGTGGTCAACAACCGACCACTGGAC
GGTGGTACGCGTTGTGGACTCTATAATACTTCAATATATTTAATACTATAAAAAAAAAAAAAAATTTAATATATTT
AAGAGATTTTTTTTGGTCAAGTAGACTAGTGGTTAGAATTCCCCTTTTTTCAAAGCGAATAAGTGGGGTGTCCGG
GTTCAAATCCGGACCCCTGCATATAATAATGCATATCTTTTCCAACTGAGCTATGCTCACCGGAATTATATTTAA
GAGATTTATTGTCGTAGTATGTTTATTTTTTTCATTTTTGAAAGGAGGCATTGTTATAGTTTAAAAAATCATAAA
AAAGAAAAAAAATTGATTACGTTAGAAACTTTACAAAAGACTGATGTAATCCAAATTAGTTTTGCCAAAATACTA
AAAGTGTACATTTTCTTCTTCTAAAAGTTTGTCTTGTCACAAAAGAAAAAAGTTTGACATAGAAAATGAGAATTC
ATTTTTTTTAGTATCATTTCTAATAATTAAATTCAACTGAAACAATTTCCAAGCCTATATTCGTTTTGAAATTCT
TCGTCTTTCGTCAGATTTATTACTTTTTTCTTTTGTCGTTGCTCATTAGTTTATTTATTTATTATTTTTCATGTT
GACTTTTGCATGGAATACAACTTCTTCAATTTCTCAAATTGAAAGTTTGTGTGAACCTGTGAATTATGTGCAAAG
ATGATTAAGCATTTCACTCATATTAGTATACTTATTAATTAATTTATACTTAATGAATAAGTATACGTAAATTCT
CAATAAGGTTGTGGTTCTCTTAATTTTTTAATATTAAAAGAAAAATAGCTTTCATTTTCATAATGATTTTTCTTA
AAGGATTCATTTTCTTAATGATGTTATTTTGCTTTTAATATAAAGCTCTCATTGAGCCACGGTGTATATATATAT
ATTCCTAATAAGTAAACGTCCCATAAATGATATTTGTACAACCATTTTGTAATAACTTTTGTGACAACTTTCTCT
CTCATACTCACATATGTTTTCATTTTATCTTTCTATTGCTTTGATATTTGTGCTAAAACTTATTTTTTTCTTTATA
AATTTATGGTTGTCAAATAAATTGTCTATCAAAAGGTTATTCAAATAACACACCTCTTTTATCAATATACACCTC
ATCCTTAAAAAATAAAATCAATATACACCTCATAAAAAAGAACTACAATATGCCAAAAATAGCAAAAGTTAAACC
ATCCCTTTAAAGAATTTTACTCTCCCAAGTCTTAACACTAGTATTAATCAAGCAGACAGCTATAATTACATATGT
TTAGCTGTTTGTACCAAAAATATTAATATTGTTGGTTTTGAATTCCGGATAAATGTTCTGAGTCATAGGTATGCC
CTTAACCCTAATGAATCCCGGAGCCCAACCCATATAAACTTTATCTTATAAAAAAATAACTCTAGAAGTATATGA
AAATTTTGTGATATTTTTCATTTAACAAGACAATATTATTTAAGGCCAATGATAAAACATATAATTTTGAAATGA
TGAATTATGTGATATTTTCCATAGATAACTAATCTATTATTGAAATAATTGTAAATTATGATTATTGTTTGTGAG
ATGCCTATCATTTAGTTTTATTGATCGCCTATAATTTTTTCCGATGATAGTTAAGTCTAGTAGAATGAGTTAGTC
TCCTACAATTTCGCAAACCACAATTTGATTACTCATTATATATAGTGGCACCCATATTTAGTGTCCTATTTCTAC
TCAAATAAGATTGCCTCTAGATTGCCTTTCATTTCATCACCAAGGAAAAAATGGTGCTCCAAATATTTAGGAAGT
GCACAAGTAAATGACTAATTAGAACAAGGTGCACCACAATTAGATTAGATACAACTACAAACTTTAAAGGTTGTA
TATATTGAAGAGATTTGATGCATAAACCCTAAAAATATATTTGATTATACCCAAAAAAAAAATCATATAGAAATT
GATTGTATTGGATTGGATTGGAGTACTCATTGTGCTCCTTTATCCAATAATCTTAATTTGTTCTTCAAATTAAGG
GAGATGTTTTATTTAAATCTCACAAAACTCATTGATCAATTTTGTTGTTTGGTATTGATGCAAAAGTGGGGTCAC
AAGATATCCCAAGATGTTCTCTTTTGTCATAGTTCTTCTTGCAAAATCAGAATCTTAATCTATCTTTTGGGTTAC
ATGGTATCTTTGTCTGGTCATCAAAACTTAACTACTCCTTGCTAGAATTTTCTCCATTTTTTTCTTATATGCTAA
ACACCCAATTCAGATCTTAAATCATGATTTGGTTTCCACAATGGAATTTTTGGTGTTTATTTGGCATTGGATTTG
ATTAGTCGTAGGTTGAGAATAGTAGAATTGTTTTTTCAAGAACTCAACCACCTGTAAATTTAATAAACAAGTATG
AAACCATAAATTAATTAAGCCACAAACAAAGTTATGAACAATCTGCTCCATTAAAACAAAGGTTATTTTATTTTT
TTATAAAATGTTGAAGTAAGTTGAACCCGAAGAGTTGCGAGGATCAAGAAAAATTTCAAGATTCAGAGACGACAA
ATACAAATACTTACATATTATCTATTAACGATGTTCTCAAACTAACAACGAACATCAAACTCAGGTCTTTGAAGA
ATCTGAGCGGAATCCTTATCAACGAAGATCAAGACAAATTTCAAGATTCATAGACCACAGATACAAGTACTTACA
TATCATCTAATAACGATGTTCTTAAACTAACAATGAACATCAAACCCAGATCTTTGAAGAATCTGAGCGGAATCC
TTATCAACTTCACCAAACAAAGTTGGTAATTTGTAAGCAAAATTAGGGAACTATAATAGCAACTTAATCAAAACA
TCCATCTCCAAACAACCACCATATAATTTCCGTGTGGATCTGTCACAGTTGTCACAAGTAGCAACAGATAAACTA
TGTGAAATTAAATTAAATTAAAGCTTAAAAGGTAAAGCTTTGATTTGCTTACACTACAAAATCAATAATTTATAT
TATATACATTTTATGCATTATTTATGCTGAATAAGCCAGCCAAGTTGAAAAGGGACCTTTTTTCCTTGTCTTTAA
GCCTCAAAGCCTATATATAGAGAAATTTCACACAAACTTTTCATTCAACTCTCTCTCACCCATTTTCTCTCTCTA
CCTATCTAACTACCTTCTCTCTCTTTCCCCTCCTTCTAAAAATAGTTAGT

MtACS3-3`UTR

TTTTTACCATCACTCTACCATGATTAAATAGTATTTTTTTAGGGTCAAATCAATCAGAAGAGTCAGAGTTTGAAT
CATGACAAAAATAATCATTTGTCAAGTTTTACTTATCTCCCAAACGAATTCCGATTACCAGTTTCTTTCGCCTTG
AAATGGGAGGGATAAGACCAAAACATTAGAGTCTTTGTTTATTTATTTTTATTTTTTTTCAAATGGTTGAGGCCA
TTTTGGTATATTCTTTTAAAAGCACATAATGTAAAGGTGAATATTCAATTGAAGTGTATCAATCCCTTGTTTGCT
TGCTTAATTTACTTAAGCAAGTTTTTATTACAAAATTAATCTTTTGTATTATTTTGTTTTATAATGAATGAAATT
TGTTACTATTAAATTATGTGTCTTAATTACTACATTATTTGTATGATGAGTTAGTATAGTGTCTATATCATCTTG
AACATCTTGATTGGTTGTGTAGTAAAAAATTAATCCTTATTGAATGAGTATTGGTCTTGAATTTATCAACATCAT
TTGACTTAGTAATTACTGATTTAATTTCAATTCATATCATAGTTGACTTAGAGTTTGGAGTTTAAAGTACGTAAC
AGCTAGCAAAACACACCAACCAGTTATAGCTTTCAGCTTCATGAAAGGGTTGGAAACTTGGTATGAATAAAATAT
TAACATGCCTCAATCCCTACAGTACATGATGATGTGCATATTTTTAATTCAAGTCTAATTGCCATGTTTATTGGA
TAATTAGTAGTATATAAGTATGATTTGATTATAACATTGTAATTAATTATAAGTATAGTATAAGTATATACTTTG

TATTAATTCCTATAGTAGTGTCGGGAGATATTCTAGAACAAATTAAAAATGGTCCACCCCACGTGATGTACACTG
CTTGAATTTATATTTGTATATATAACTGCATCTCTTTGTTGTTGTTGTTGTGTTGTGTGTGTGTGAGGAAAATGA
TGTTGATATTGATTGATATTAAAAT

MtACS10p

GACCTATCGTGATTATGGTAAACACTTGAGATCCAACTCACTAAACGATAACAATATGATTTAAATTCATGTAAT
TGCCATCAAATTTATATGAGATTAACATGATTTAGTGAAACTGATGTGAATTAGGTTCAGCAGTGTATTGTGTGT
GTGGAAAGTGGAAACCATAGTATTCAGTTCCACAATTCTGTCACCGACCAACAAGGGTAAGCAGTGCATATGGCG
CCAACAACACGGCAATAATTCTATTCCAATTGTGAGTATCTTTGTTAGGTTAGTACTTAGTGGTTTGGTTACACT
CTCTTTTCATTTCATTTCATGGGATGGGACATAATGCCACTTGTGTTTTCATTTAATGCTCACAGCACAAAGTCA
CACTCTTCTTCTTCCAACAATTCCACTCTCAATTAATACTACGTGTCAAAAATTGAAACTGTGACCCACCTTTGT
AGACAGAAATAAATGAATACGTCCCATGAAAATCTATGGGTTGGTCTGGTGTAGGGTTGTACAAATCCCTCTCAT
GAAGGGCATCACCCTAATATAGAAAAGATATGAGGACAAAAGGTTGTCCAAGAACATTTTTTTTACGATCGAGTA
AATACCAATATGGACTCTATCTTTATAAGTTATTATCAAATTAGTTTCTCATTTTATTAATTTTTTAAAATAGTC
CCTCAAAAGTTTCTCAATTATATCATTGCCTCTATGTTTTTGTCATATGACGAGGAATTTAATTGACTTAATTGA
AAAACTTTTGACAAAGACATGAACTATTTTTAAATATTCTCAAAAGCTTCCAATTAGATCCTTTTCTATTTGTTT
TTGTCACAAGACGATAGACTTAATTGAAAAATTTTTGACAAAAATAAAAACTAATTTAAAATATTAATAGAGTCA
GAGACTGATTTGAAAATTACTTACAAAGATAGAGAGCAAATTAACAGATTACTCATTTTTTTACTTATACATTTG
ATAATATATATTATGAAAAAAAGTCTCAAATAATTTTATCTATATTTAATTGGAGTTTTTGATATATTTAATTTT
CACCAAATTAAATATAGATATTGAATCACTATCTTAACTTGCTTAAGCACTCAATCAAATTTTTCATCAGCCTTT
TTGTCATGGTAGGTGGCATAATGCTTTCTTCATTCTTTCAATTTATTTTTTTCCAATACATATATATTTATGATT
TAATTTGTGTAGGCCGATAATTGTAAAGCTTTTACACATTTAAATTACTCTACAATGTCCTCTTTTTAGGTTGTT
CCTAAAACATCTCTACACATATCTATTTAACATTAATTTGATTAGATGCATATAAAAATGTTTTAGACTTTTTGT
GCATAGAAATGAGTCTCTATATTTACATATTGATTTAAGTATTTGTCAATAAAAACTATTATATATTATTTTAGT
GCGTGTTGTGTAGATGGATTACTAACTACGAAGATATCTGAATCTACATTCATATCCCCCTCATTTTTTCAAATA
ATTATTGGTGCTCATGCCAATATCTATAATGGTTTTTTACTCGACCAATTAATATAATTTGGTTTGCAGGTATCA
AATAAATAAATAAGGCAACTTCTTCTGCGGTATACCTCACAAATTGGGGTGTACCGGTACTCCTTGCTTCATAAA
TTGATAAATTAACGATCATTTTAGAAAGCAAAGAGCTATTTGTCAAGGTTATTCTTTAGAAAACATCATTTCATA
AGAAAAAAAAACATAATTTCATTGTTTATAAGAATCATATTAAATTTTTAACATTTTCTCATAAAAAATAATCAA
TAATCTTCATTATGAGTAATAAAAATTCAAAAGAATTTAATTTAATTTCATCGACATTTTGGTAAATAAGATTTA
ATTTTTATAATTGTCAATGATAGATTTCTTCAAAAATTCATCTAATTTAATTAATAATTTAACAATTTGGTGTAT
CGGTACACTCAAATATTTGGGTGTACCATATAATCCACTAGTCTCTGCCCTATAAGACACTCTCAAATAAACTAT
AAGATTATGAGTATTTTAAAACAATCTCTATTCTCTATATTTAACCACTTACTATTAATTTTACATTAATTAAGT
CATTAAATTTTAACAATTTACTATCGATTTCCCGCAAAAAAAAAATTAATATCAATTTAATTCATAAATTTATCA
TTCACCATCAAATTAATCATACCATATTTTTTAAAGAACTAATATAAGTAAGATTTGATAAAGTCAAAAATATTT
TTAAAATATTCATAATGCTAATTACCTATTTGAGAACGTCCTATAAAGTCTGGAATTAATTTGTTGATTTAGAGA
CTAATCTTCATCTCTGCACCCCTGTCCAATCCATAACTCTTTCCCATCTTTATGACCCTGGCCACATCAACTTCC
ACCAACAAATAGATAACAGATCAGAGCGTACGCAAAAATACAAATAAGTAGGTGAAACTATATGTCAATTGTATA
ATAAATAACAATTATACTATTTTTTTAAATAACAATTATACTAATATTATTATACTTTTTTTTTGGGTGTACATA
ATAATATTATTATACAAATAAAAAGTAATATTCCGTCTTATGATTATTAAAAAAAAACAGTATTATGCATAGGAA
CGTATACAACTGTAGTACAACACAAAACCTGAATACAAAGGTCATCACAAACTTGAAGTCTAATATTTTTCTCCC
ACTTTGTTGACACAAAAACAAAATACAAGTGGCATTTTGCTATTTGAACGACAATTATTACTAGTTATTGTCTGC
CTACCAGCATCTGTCTAATTTTCAACTGAAGCTGCTGGCGTGGCATCATAGCTGAAAATGCTGCCAGAGTCCAAA
AATAATTGCTCATTCACTATTATTAGGAGATATCGTTTAATCGATTGATTAGACTCAAATGATTAAAGAGTTTTT
CTTAAAAGGAAGTGTTAGGTTTTGTTGGAGAAACTCTTTAAAGATTTAAATTTTTTTATTTTTTATAAAAGGATA
AATATTTTGATTTTCAATGCATTAATTACATATATTTTCATAAAAAATTATCATTTAAAAGTATTAAAGAGTGTC
CGGAGAAACTCGTCCATATTTTCCAAATAATTTTTGGATTTTGGTTTCCTATGACCCATTTTTCCTAAAAATTAA
AGGCTTAACAAAAAAATATATTTGCTAGCTAGACTCTTACGCTCTCACCATTACCTTCATGTGAACCTTCCTTTT
TTATATTTGGACTAACTAAAAAAATATTCTTATGTCTGAAAATCAAAATATATTTGTGCATATGCGCATGCTATA
AATAACCACCCCATACTTCATTCCTACACATTGCAATCTCTTCTAACTTATTCTTACACCTTCAAACATAAGAAG
AATTCCCACACAAATTCGCTTCTTCCTCGTACTTTTTTGTTTTTGTTTTTGAATCTCATTGATTTCATCACATTT
TCTATTAAATTAATTAATATTTTAGACTATAGTAATTAATA

MtACS10-3`UTR

ATTATTTAGCATATAGAAGAAGTAATAACTGATAATTTTTTTTTTTTTTTTTTTGGTTTGTTGATTTATCAGTTT
GGGAAACCACATGTTGTGTTCCTTTAATGTTGGGGGCATTTTTTCTTTTTTCACTTACTGAATCACATTGGTTTG
TGGTGAAAGAAGTTCATAGAATTTTTAAGGTCAATGATGAATGAATTGTTAATAAACTTCTTCAATTATTCTACA
TGTTTTATTGTTGTTTTATAAACTGTTAAAGATAAATCAACTTTAGATTTGTACTAGTATGATAATGATACATAT
CAAATAGTATAAAGGAATTGAAGTGGATGTATGAGTAATTCACTAAAGATAACAAGACTACTGAATAAACATTCA
ATTTTTTATTTTTGGAATAAAGAAATTTACAAACAAGAAATTGCAGGGAGATTTACTCAAAAATATTTGTATTGA
AAGTGATTCAAGTACTACCGTCCAACTTGCAAACCATCACCATGTCCACATGATTCACTTTCACGGTTTCTTTGC

TATAAGATGATGCAAAGATTCAGTCTGGTCAAAAAATTGTAAAATACTATTAAAACGCAGTCTCAATTTGGATCC
GTTGAATGAAAATTTCACAATTCATATTCCAAATTCATAGTTTGAATATGACTTAAGGTTGCACAAATTGAATAT
GAGCAGTTTCATTTGCTAAAAAAATAAGGAATCGGAATTCGGACATGATGACAACTCTTACGGTACCCTATATGA
TTTACACATTGGTCACGAGATTGGCCAATATAGGGGCAACGAACTGGATAATACATAGGACAAAAGACTAAACAA
AAACATAAATTCTTGTCCTTAATTTTTTTTTTTATTTGGAATACAGTCTACTTGGTTGGAAGGTTATGTTTGACT
TCAACTCAAGATTTTTTTTCTTTTTCTTGAAGAGGCTCAAGAAATATTTCTTGAGTTGAGACACGTGAAATAGGT
CGCGGAGGCAATGTTAACTGATACC

Supplementary Table S6: Statistical information.

Figure	Comparison / Variable	Test	Factors	n	df		p-value	Notes
Fig. 2E	Gene expression	t-test	LCO aplication	2	2	*MtACO1*<br>*MtACO2*<br>*MtACO3*<br>*MtACO4*<br>*MtACO5*	0.30<br>0.23<br>0.45<br>0.05<br>0.79	paired, equal variance
Fig. 2F	Gene expression	t-test	LCO aplication	2	2	*MtACD*	0.78	two-tailed distribution, equal variance
Fig. 2G	Gene expression	t-test	LCO aplication	2	2	*MtACS1*<br>*MtACS2*<br>*MtACS3*<br>*MtACS4*<br>*MtACS6*<br>*MtACS8*<br>*MtACS9*<br>*MtACS10*	0.008<br>0.09<br>0.048<br>*n.a.*<br>0.42<br>0.02<br>*n.a.*<br>0.003	two-tailed distribution, equal variance
Fig. 3A	Gene expression	t-test	LCO aplication	3	4	*MtACS3*	4.80E-05	two-tailed distribution, equal variance
Fig. 3B	Gene expression	t-test	LCO aplication	3	4	*MtACS10*	0.016	two-tailed distribution, equal variance
Fig. 4A	Nodulation	ANOVA	Transgenic	>10	df1=2, df2=30	Between genotypes	0.0001	One-way
	Nodulation	Tukey's HSD	Transgenic	>10	df1=2, df2=30	EV vs *acs3i*<br>EV vs *acs10i*<br>*acs3i* vs *acs10i*	0.93<br>0.0002<br>0.0001	p-adj
Fig. 4B	Nodulation	ANOVA	Transgenic	>10	df1=2, df2=45	Between genotypes	0.003	One-way
	Nodulation	Tukey's HSD	Transgenic	>10	df1=2, df2=45	EV vs *acs3i*<br>EV vs *acs10i*<br>*acs3i* vs *acs10i*	0.005<br>0.98<br>0.004	p-adj
Fig. 4C	Nodule initiation	t-test	Genotype	>19	43	R108 vs *Mtacs3-1*	1.28E-08	two-tailed distribution, equal variance
Fig. 4D	Nodule initiation	t-test	Genotype	>19	43	R108 vs *Mtacs3-1*	0.32	two-tailed distribution, equal variance
Fig. 4E	Nodule initiation	t-test	Genotype	9	16	R108 vs *Mtacs10-1*	0.01	two-tailed distribution, equal variance
Fig. 4F	Nodule initiation	t-test	Genotype	9	16	R108 vs *Mtacs10-1*	0.007	two-tailed distribution, equal variance
Fig. 5A	Number of infection treads	ANOVA	Genotype	>24	df1=2, df2=72	Between genotypes	0.0001	One-way
	Number of infection treads	Tukey's HSD	Genotype	>24	df1=2, df2=72	R108 v *Mtacs3-1*<br>R108 *vs Mtacs10-1*<br>*Mtacs3-1 vs Mtacs10-1*	0.0001<br>0.42<br>0.0001	p-adj
Fig. 6A	Nodule position	ANOVA	Genotype	>18	df1=2, df2=55	Between genotypes	0.004	One-way
	Nodule position	Tukey's HSD	Genotype	>18	df1=2, df2=55	R108 v *Mtacs3-1*<br>R108 *vs Mtacs10-1*<br>*Mtacs3-1 vs Mtacs10-1*	0.008<br>0.55<br>0.05	p-adj
Fig. 7A	Gene expression	t-test	LCO aplication	3	4	*MtACS10* mock vs LCO<br>*MtCRE1 mock vs LCO*	0.027<br>0.017	two-tailed distribution, equal variance
Fig. 7E	Nodulation in *ACSox* lines	ANOVA	Transgenic	>10	df1=2, df2=27	Between genotypes	0.005	One-way
	Nodulation in *ACSox* lines	Tukey's HSD	Transgenic	>10	df1=2, df2=62	*GUS vs ACS3ox*<br>*GUS vs ACS10ox*<br>*ACS3ox vs ACS10ox*	0.007<br>0.01<br>0.18	p-adj
Fig. 8C	Nodulation	Fisher's exact test	Genotype	>10	-	A17 Z1 vs *skl* Z1<br>A17 Z2 vs skl Z2<br>A17 Z3 vs skl Z3<br>A17 Z4 vs skl Z4<br>A17 Z5 vs *skl* Z5	0.598<br>1<br>2.19E-5<br>3.40E-5<br>4.58E-4	
	Nodulation	Cochran's Q test	Zone effect	>10	4	Zones in A17<br>Zones in *skl*	0.0001<br>0.18	
Fig. 8D	Root lenth 1 D.A.G.	t-test	Genotype	>15	30	A17 vs *skl*	0.0009	two-tailed distribution, equal variance
Fig. 8E	Root lenth 15 D.A.G.	t-test	Genotype	>15	30	A17 vs *skl*	1.07E-11	two-tailed distribution, equal variance
Fig. 8F	First root hair formed	t-test	Genotype	>5	9	A17 vs *skl*	0.005	two-tailed distribution, equal variance
Fig. 8K	Root hair length	t-test	Genotype	>5	11	A17 vs *skl*	1.04E-07	two-tailed distribution, equal variance

Supplemental figures

Fig. S1A	Gene expression	t-test	LCO aplication	3	4	*MtACS1*<br>*MtACS2*<br>*MTACS4*<br>*MtACS5*<br>*MtACS6*<br>*MtACS7*<br>*MtACS8*<br>*MtACS9*	0.003<br>*n.a.*<br>*n.a .*<br>0.938<br>0.554<br>0.992<br>0.037<br>-	two-tailed distribution, equal variance

Fig. S1B	Gene expression	t-test	LCO aplication	3	4	*MtACO1*<br>*MtACO2*<br>*MtACO3*<br>*MtACO4*<br>*MtACS5*	0.94<br>0.64<br>0.46<br>0.23<br>*n.a.*	two-tailed distribution, equal variance
Fig. S1C	Gene expression	t-test	LCO aplication	3	4	*MtACD*	0.18	two-tailed distribution, equal variance
Fig. S3B	ACC levels	t-test	LCO aplication	6	10	A17	0.96	two-tailed distribution, equal variance
Fig. S5	Gene expression in RNAi	ANOVA	*ACS2*	5	df1=2, df2=12	Between genotypes	0.16	one-way
	Gene expression in RNAi	Tukey's HSD	*ACS2*	5	df1=2, df2=12	A17 *vs acs3i*<br>A17 *vs acs10i*<br>*acs3i vs acs10i*	0.55<br>0.15<br>0.11	p-adj
Fig. S5	Gene expression in RNAi	ANOVA	*ACS3*	5	df1=2, df2=12	Between genotypes	0.04	one-way
	Gene expression in RNAi	Tukey's HSD	*ACS3*	5	df1=2, df2=12	A17 *vs acs3i*<br>A17 *vs acs10i*<br>*acs3i vs acs10i*	0.045<br>0.28<br>0.03	p-adj
Fig. S5	Gene expression in RNAi	ANOVA	*ACS5*	5	df1=2, df2=12	Between genotypes	0.56	one-way
	Gene expression in RNAi	Tukey's HSD	*ACS5*	5	df1=2, df2=12	A17 *vs acs3i*<br>A17 *vs acs10i*<br>*acs3i vs acs10i*	0.82<br>0.46<br>0.61	p-adj
Fig. S5	Gene expression in RNAi	ANOVA	*ACS7*	5	df1=2, df2=12	Between genotypes	0.43	one-way
	Gene expression in RNAi	Tukey's HSD	*ACS7*	5	df1=2, df2=12	A17 *vs acs3i*<br>A17 *vs acs10i*<br>*acs3i vs acs10i*	0.98<br>0.60<br>0.63	p-adj
Fig. S5	Gene expression in RNAi	ANOVA	*ACS8*	5	df1=2, df2=12	Between genotypes	0.14	one-way
	Gene expression in RNAi	Tukey's HSD	*ACS8*	5	df1=2, df2=12	A17 *vs acs3i*<br>A17 *vs acs10i*<br>*acs3i vs acs10i*	0.17<br>0.64<br>0.28	p-adj
Fig. S5	Gene expression in RNAi	ANOVA	*ACS10*	5	df1=2, df2=12	Between genotypes	0.01	one-way
	Gene expression in RNAi	Tukey's HSD	*ACS10*	5	df1=2, df2=12	A17 *vs acs3i*<br>A17 *vs acs10i*<br>*acs3i vs acs10i*	0.21<br>0.02<br>0.01	p-adj
Fig. S6	Nodulation on RNAi lines	ANOVA	Non-transgenic	>10	df1=2, df2=30	A17 *vs acs3i*<br>A17 *vs acs10i*<br>*acs3i vs acs10i*	0.43	one-way
	Nodulation on RNAi lines	Tukey's HSD	Non-transgenic	>10	df1=2, df2=30	A17 *vs acs3i*<br>A17 *vs acs10i*<br>*acs3i vs acs10i*	0.53<br>0.995<br>0.47	p-adj
Fig. S8	Root lenth 15 D.A.G.	ANOVA	Genotype	>14	df1=2, df2=54	Between genotypes	0.0001	one-way
	Root lenth 15 D.A.G.	Tukey's HSD	Genotype	>14	df1=2, df2=54	*R108 v Mtacs3, R108 vs Mtacs10, Mtacs3 vs Mtacs10*	0.0001<br>0,80<br>0.0001	ap-adj
Fig. S8	ACC levels Zone 1	t-test	Genotype	5	8	*R108 v Mtacs3, R108 vs Mtacs10, Mtacs3 vs Mtacs10*	0.0002<br>0.26<br>0.0003	two-tailed distribution, equal variance
Fig. S8	ACC levels Zone 2	t-test	Genotype	5	8	*R108 v Mtacs3, R108 vs Mtacs10, Mtacs3 vs Mtacs10*	0.46<br>0.0003<br>0.0007	two-tailed distribution, equal variance
Fig. S10A	Nodule initiation	t-test	Genotype	>22	51	R108 vs *Mtacs3-2*	1.01E-05	two-tailed distribution, equal variance
Fig. S10B	Nodule initiation	t-test	Genotype	>22	51	R108 vs *Mtacs3-2*	0.42	two-tailed distribution, equal variance
Fig. S10C	Nodule initiation	t-test	Genotype	>15	31	R108 vs *Mtacs10-2*	1.01E-07	two-tailed distribution, equal variance
Fig. S10D	Nodule initiation	t-test	Genotype	>15	31	R108 vs *Mtacs10-2*	3.98E-07	two-tailed distribution, equal variance
Fig. S11	Number of infection treads	ANOVA	Genotype	>24	df1=2, df2=76	Between genotypes	0.0001	One-way

	Number of infection treads	Tukey's HSD	Genotype	>24	df1=2,<br>df2=76	R108 v *Mtacs3-2*, R108 *vs Mtacs10-2*, *Mtacs3-2 vs Mtacs10-2*	0.0001<br>0.60<br>0.0001	p-adj
Fig. S12	Nodulation on *ACSox* lines	ANOVA	Non-transgenic	>15	df1=2,<br>df2=49	Between genotypes	0.06	One-way
	Nodulation on *ACSox* lines	Tukey's HSD	Non-transgenic	>15	df1=2,<br>df2=49	*GUS vs ACS3ox*<br>*GUS vs ACS10ox*<br>*ACS3ox vs ACS10ox*	0.06<br>0.88<br>0.09	p-adj
Fig. S13C	Root hair length	t-test	Genotype	>6	df1=2,<br>df2=18	*R108 v Mtacs3-1*<br>*R108 vs Mtacs10-1*<br>*Mtacs3-1 vs Mtacs10-1*	2.7E-06<br>0.16<br>6.66E-05	two-tailed distribution, equal variance
Fig. S14A	*MtACS3* expression	t-test	LCO aplication	3	4	*Zone1- vs Zone1+*<br>*Zone2- vs Zone2+*<br>*Zone3- vs Zone3+*<br>*Zone4- vs Zone4+*<br>*Zone5- vs Zone5+*	0.56<br>0.001<br>0.74<br>0.36<br>0.88	two-tailed distribution, equal variance
Fig. S14B	*MtACS10* expression	t-test	LCO aplication	3	4	*Zone1- vs Zone1+*<br>*Zone2- vs Zone2+*<br>*Zone3- vs Zone3+*<br>*Zone4- vs Zone4+*<br>*Zone5- vs Zone5+*	0.60<br>0.001<br>0.01<br>0.16<br>0.30	two-tailed distribution, equal variance
Fig. S14C	ACC levels at 2 hrs	ANOVA	LCO aplication, zonation	5	df1=1,<br>df2=40	Treatment	0.515	2-way
	ACC levels at 2 hrs	ANOVA	LCO aplication, zonation	5	df1=4,<br>df2=40	Position	2.20E-08	2-way
	ACC levels at 2 hrs	ANOVA	LCO aplication, zonation	5	df1=4,<br>df2=40	Treatment × Position	0.955	2-way
Fig. S14C	ACC levels at 2 hrs	Tukey's HSD test	LCO aplication, zonation	5		*Zone1+ vs Zone2+*<br>*Zone1+ vs Zone3+*<br>*Zone1+ vs Zone4+*<br>*Zone1+ vs Zone5+*<br>*Zone2+ vs Zone3+*<br>*Zone2+ vs Zone4+*<br>*Zone2+ vs Zone5+*<br>*Zone3+ vs Zone4+*<br>*Zone3+ vs Zone5+*<br>*Zone4+ vs Zone5+*<br>*Zone1+ vs Zone1-*<br>*Zone1+ vs Zone2-*<br>*Zone1+ vs Zone3-*<br>*Zone1+ vs Zone4-*<br>*Zone1+ vs Zone5-*<br>*Zone2+ vs Zone1-*<br>*Zone2+ vs Zone2-*<br>*Zone2+ vs Zone3-*<br>*Zone2+ vs Zone4-*<br>*Zone2+ vs Zone5-*	0<br>0<br>0<br>0<br>0.9982<br>0.9894<br>0.9901<br>1<br>1<br>1<br>0.9996<br>0<br>0<br>0<br>0<br>0<br>1<br>0.999<br>0.9892<br>1	adj p

	ACC levels at 2 hrs	Tukey's HSD test	LCO aplication, zonation	5		*Zone3+ vs Zone1-*	0	adj p
						Zone3+ vs Zone2-	0.9996	
						Zone3+ vs Zone3-	1	
						Zone3+ vs Zone4-	1	
						Zone3+ vs Zone5-	0.9999	
						Zone4+ vs Zone1-	0	
						Zone4+ vs Zone2-	0.9963	
						Zone4+ vs Zone3-	1	
						Zone4+ vs Zone4-	1	
						Zone4+ vs Zone5-	0.9991	
						Zone5+ vs Zone1-	0	
						Zone5+ vs Zone2-	0.9966	
						Zone5+ vs Zone3-	1	
						Zone5+ vs Zone4-	1	
						Zone5+ vs Zone5-	0.9992	
						Zone1- vs Zone2-	0	
						Zone1- vs Zone3-	0	
						Zone1- vs Zone4-	0	
						Zone1- vs Zone5-	0	
						Zone2- vs Zone3-	0.9998	
						Zone2- vs Zone4-	0.9962	
						Zone2- vs Zone5-	1	
						Zone3- vs Zone4-	1	
						Zone3- vs Zone5-	1	
						Zone4- vs Zone5-	0.9991	
Fig. S14C	ACC levels at 4 hrs	ANOVA	LCO aplication, zonation	5	df1=1, df2=40	Treatment	0.369	2-way
	ACC levels at 4 hrs	ANOVA	LCO aplication, zonation	5	df1=4, df2=40	Position	2.20E-08	2-way
	ACC levels at 4 hrs	ANOVA	LCO aplication, zonation	5	df1=4, df2=40	Treatment × Position	0.918	2-way
Fig. S14C	ACC levels at 2 hrs	Tukey's HSD test	LCO aplication, zonation	5		*Zone1+ vs Zone2+*	0	adj p
						Zone1+ vs Zone3+	0	
						Zone1+ vs Zone4+	0	
						Zone1+ vs Zone5+	0	
						Zone2+ vs Zone3+	0.9978	
						Zone2+ vs Zone4+	0.9846	
						Zone2+ vs Zone5+	0.9882	
						Zone3+ vs Zone4+	1	
						Zone3+ vs Zone5+	1	
						Zone4+ vs Zone5+	1	
						Zone1+ vs Zone1-	0.9574	
						Zone1+ vs Zone2-	0	
						Zone1+ vs Zone3-	0	
						Zone1+ vs Zone4-	0	
						Zone1+ vs Zone5-	0	
						Zone2+ vs Zone1-	0	
						Zone2+ vs Zone2-	1	
						Zone2+ vs Zone3-	0.9955	
						Zone2+ vs Zone4-	0.9531	
						Zone2+ vs Zone5-	0.9655	

ACC levels at 2 hrs	Tukey's HSD test	LCO aplication, zonation	5	*Zone3+ vs Zone1-*	0	adj p
				Zone3+ vs Zone2-	0.9999	
				Zone3+ vs Zone3-	1	
				Zone3+ vs Zone4-	1	
				Zone3+ vs Zone5-	1	
				Zone4+ vs Zone1-	0	
				Zone4+ vs Zone2-	0.9987	
				Zone4+ vs Zone3-	1	
				Zone4+ vs Zone4-	1	
				Zone4+ vs Zone5-	1	
				Zone5+ vs Zone1-	0	
				Zone5+ vs Zone2-	0.9991	
				Zone5+ vs Zone3-	1	
				Zone5+ vs Zone4-	1	
				Zone5+ vs Zone5-	1	
				Zone1- vs Zone2-	0	
				Zone1- vs Zone3-	0	
				Zone1- vs Zone4-	0	
				Zone1- vs Zone5-	0	
				Zone2- vs Zone3-	0.9998	
				Zone2- vs Zone4-	0.9924	
				Zone2- vs Zone5-	0.9953	
				Zone3- vs Zone4-	1	
				Zone3- vs Zone5-	1	
				Zone4- vs Zone5-	1	

n.a. not analysed

Breakspear, Andrew, Chengwu Liu, Sonali Roy, Nicola Stacey, Christian Rogers, Martin Trick, Giulia Morieri, et al. 2014. “The Root Hair ‘Infectome’ of Medicago Truncatula Uncovers Changes in Cell Cycle Genes and Reveals a Requirement for Auxin Signaling in Rhizobial Infection.” *The Plant Cell* 26 (12): 4680–4701.

Gómez-Fernández, Germán O., Robin van Velzen, Jeong-Hwan Mun, Douglas R. Cook, Wouter Kohlen, and Estíbaliz Larrainzar. 2025. “Ethylene Biosynthesis in Legumes: Gene Identification and Expression during Early Symbiotic Stages.” *Journal of Experimental Botany*, February. https://doi.org/10.1093/jxb/eraf069.

Schiessl, Katharina, Jodi L. S. Lilley, Tak Lee, Ioannis Tamvakis, Wouter Kohlen, Paul C. Bailey, Aaron Thomas, et al. 2019. “NODULE INCEPTION Recruits the Lateral Root Developmental Program for Symbiotic Nodule Organogenesis in Medicago Truncatula.” *Current Biology: CB* 29 (21): 3657-3668.e5.
